# Sensitive Fluorescence Quantitation and Efficient Free Radical Characterization of *N*-Glycans via LC-FLR-HRMS/MS with a Novel Fluorescent Free Radical Tag

**DOI:** 10.1021/acs.analchem.4c06294

**Published:** 2025-03-25

**Authors:** Rayan Murtada, CJ Szafranski, Maria Tevletidis, Shane Finn, Wilthon Gilles, Tabia Tahsin, Jinshan Gao

**Affiliations:** † Department of Chemistry and Biochemistry, Montclair State University, 1 Normal Avenue, Montclair, New Jersey 07043, United States; ‡ Sokol Institute of Pharmaceutical Life Sciences, Montclair, New Jersey 07043, United States

## Abstract

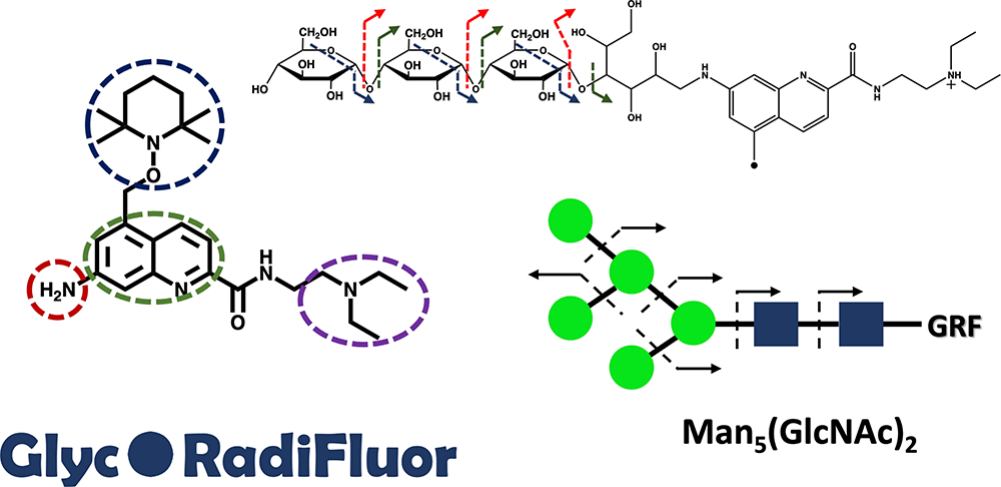

Glycans are some of the most difficult biomolecules to analyze owing to their branching tendencies as well as their regiochemical and stereochemical diversity. Yet, the correlation between various pathological states and glycan quantity or structural alterations has demonstrated the importance and urgency for the development of a more robust glycan analytical technique. Furthermore, the manufacturing and regulation of biopharmaceuticals demands a feasible and improved analytical approach toward the characterization and quantitation of glycosylations. Unfortunately, multiple commercially available glycan tags lack, in combination, liquid chromatography detection sensitivity, chemical stability and, most importantly, optimal glycan characterization capabilities. Therefore, a novel fluorescent tag coupled with a free radical approach for glycan characterization was designed and developed to help address this gap in glycan analysis. The analytical capabilities of this novel tag were assessed via hydrophilic liquid chromatography–fluorescence quantitation and ESI/MS free radical-mediated characterization by using linear glycan standards, branched isobaric glycans lacto-*N*-difucohexaose I and lacto-*N*-difucohexaose II, and *N*-glycans released from ribonuclease B.

## Introduction

The continuously evolving field of glycomics has provoked significant research in recent decades where much attention has shifted toward the development of analytical techniques for quantitating and characterizing glycans. Unlike other biopolymer molecules, such as peptides and nucleic acids, which involve the linkage of subunits via a defined stereochemical backbone that consists of amide and phosphodiester bonds, respectively, glycans can have monosaccharide subunits arranged in a branched manner via complex regiochemical and stereochemical linkages.^1^ Because glycan structures differ and are described by their types of connectivity, monosaccharide composition, and overall configuration, many constitutional isomers and stereoisomers are possible. Like other biomolecules, glycans are involved in essential functions, especially major metabolic and structural roles.^2^ Moreover, it is well-documented that glycans can vary by structure and abundance upon the onset of numerous diseases, including cancer metastasis, autoimmune diseases, hereditary diseases, pathogen-host interactions, and immune recognition.^3−7^ Recently, a correlation between neurodegenerative diseases such as Parkinson’s or Alzheimer’s and variations in *N*- and/or *O*-glycosylation architecture and abundance have also been noted.^8^ Therefore, further advancements in the field of glycomics may lead to a more prevalent use of glycans as early biomarkers for human pathological states in the clinical setting. Equally crucial to consider is the analysis of glycosylations that decorate biopharmaceuticals, requiring an analytical approach that facilitates the characterization of glycans for monitoring and regulation purposes. Even then, the glycosylation of biopharmaceuticals is difficult to control as slight condition differences in pH,^9^ cell line,^10^ dissolved oxygen,^11^ temperature,^12^ ammonia concentrations,^13^ and manufacturing mode^14^ can influence glycosylation. Thus, strides in glycan characterization methodology can have profound impact toward elucidating the presence of any glycosylations affecting the safety, efficacy, half-life, immune response, and target binding of biopharmaceuticals.^15−19^ Ultimately, the development of an improved and modern analytical technique that is capable of simultaneously quantitating and characterizing glycans from a host of sources across numerous industries would have prevalent impact.

Numerous techniques have been involved in the study of glycans, including high-performance liquid chromatography (HPLC),^20−22^ ion mobility,^23−27^ electrophoresis,^28,29^ and nuclear magnetic resonance (NMR)^30,31^ spectroscopy. For glycan structural analysis, HPLC, electrophoresis, ion mobility, and NMR spectroscopy require well-characterized glycan standards that must be pure enough, which is challenging, time-consuming, and costly to obtain. NMR data are difficult to interpret since glycans involve many carbons and protons with similar chemical environments. Nonetheless, the use of HPLC for glycan quantitation following glycan characterization via electrospray ionization/mass spectrometry (ESI/MS) is one of the most robust and optimal combinations. ESI/MS is noted for multiple dissociation techniques, minimal sample consumption, short acquisition time, high sensitivity, high mass accuracy, and high resolution. However, the ionization efficiency and fragmentation of free glycans via MS is relatively poor and challenging. Therefore, to aid in the analysis of glycans, some research laboratories have elected to develop and utilize derivatization reagents like 2-aminobenzoic acid (2-AA), 2-aminobenzamide (2-AB), procainamide, and RapiFluor-MS. Out of these, RapiFluor-MS utilizes the more sensitive quinolinyl fluorophore and possesses the greatest ESI/MS sensitivity.^33^ However, a limitation of RapiFluor-MS is that it introduces challenges to glycan analysis, particularly poor chemical stability, undesirable labeling of proteinaceous amines, and limited analysis to a single category of glycans.^32−34^ Therefore, a novel tagging reagent that addresses these shortcomings is desirable in order to improve modern glycan analysis.

There are an extensive number of MS dissociation techniques that have been used, and yet, new techniques are continuously being developed. For instance, low-energy collision-induced dissociation (CID) and infrared multiphoton dissociation (IRMPD) are commonly known to generate fragmentations via glycosidic bond cleavages.^35−40^ In comparison, ultraviolet photodissociation (UVPD)^41−43^ and higher-energy collision dissociation (HCD)^44^ have previously been shown to generate analyte fragmentations that are more information-rich. Electron-capture dissociation (ECD),^36,45−47^ electronic excitation dissociation (EED),^22,48−50^ electron transfer dissociation (ETD),^39,51−54^ and electron detachment dissociation (EDD),^55,56^ often grouped together as free radical-driven dissociation techniques, have similarly demonstrated great potential for glycan structural analysis. Meanwhile, by combining MS^n^ and CID or HCD with a tag that recruits a free radical precursor, glycan structural analysis can be feasibly, accurately, and rapidly performed.^57−60^ Besides, free radical chemistry also has attracted significant attention in the field of proteomics^61−69^ and lipidomics.^70−73^ Moreover, gas-phase ion/ion reactions that internally occur in modified mass spectrometers and are capable of charge-inverting anionic analytes have been additionally demonstrated, showing the potential for future applications into glycan structural analysis.^74,75^ Currently, experiments in both the gas-phase and condensed-phase are being performed where a reagent, which employs a free radical precursor, forms a complex with the analyte of choice, allowing for free radical-directed fragmentations to subsequently occur. However, a limitation of this approach is that it does not enable sensitive detection and quantitation of glycans.

**Figure 1:**
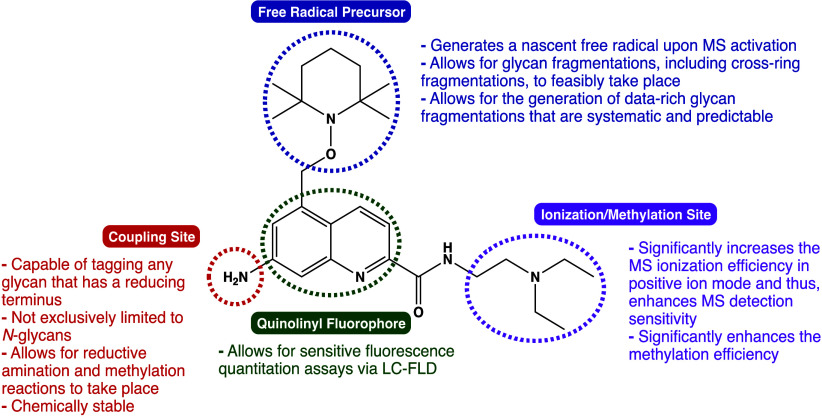
Novel fluorescence free radical tag (**Glyc**•RadiFluor) for glycan characterization and quantitation.

Previously, free radical-activated glycan sequencing (FRAGS) tags were designed to derivatize glycans at their respective reducing termini.^58,59,76^ The FRAGS enlist a free radical precursor (2,2,6,6-tetramethyl-1-piperidinyloxy, or TEMPO) which, upon collisional activation of the derivatized glycan, efficiently generates a localized, nascent, free radical. Unlike nonfree radical-directed techniques, the free radical simultaneously induces predictable, diagnostic, and systematic glycan fragmentations, heightening the quality of MS^2^ spectra while also allowing cross-ring fragmentations to occur. Furthermore, a basic pyridyl site for charge induction is recruited in the FRAGS, in addition to the hydrazide or aminooxy labeling sites which react with the glycan reducing terminus. Formerly, the first-generation FRAGS was methylated at the pyridine moiety (Me-FRAGS I) and was covalently attached to isomeric oligosaccharides with subtle differences. In brief, the importance of localizing a fixed charge via methylation is to eliminate the induction of gas-phase glycan rearrangements through a mobile proton. Using Me-FRAGS I, MS^2^ and MS^3^ collisional-induced dissociation (CID) spectra had shown its ability to accurately and rapidly distinguish among all nine isomeric disaccharides and among two isomeric tetrasaccharides.^60^ Although the FRAGS-derivatized glycans can be detected via liquid chromatography, they lack a fluorescently active moiety that would help optimize the sensitivity for liquid chromatography detection and quantitation. Moreover, the hydrazide or aminooxy groups in FRAGS generate three isomers due to the interconversion of glycan isomers upon derivatization, giving rise to multiple chromatographic signals and complicating glycan quantitation. A substitution with an amino group, however, enables the use of a reducing agent to reduce the resulting Schiff base and eliminate the interconversion of glycan isomers. With all these crucial considerations, we addressed the challenges by developing a novel fluorescent free radical tag (**Glyc**•RadiFluor) enlisting the following functionalities: (1) a free radical precursor that yields a nascent free radical that is capable of inducing systematic and predictable glycan fragmentations; (2) an ionization or methylation site that allows for enhanced ionization efficiency and methylation efficiency; (3) a fluorophore which allows for sensitive quantitation of derivatized glycans; and (4) an amino coupling site which reacts with the unique reducing termini of glycans via reductive amination (Figure 1). In contrast to older tags, **Glyc**•RadiFluor is chemically stable and analytically capable of simultaneous sensitive quantitation and free radical-directed characterization of glycans.

## Experimental Section

### Materials

For the synthesis of the **Glyc**•RadiFluor, 1,2-dimethyl-3,5-dinitrobenzene was purchased from 1ClickChemistry (Kendall Park, NJ, USA) while all other chemicals (see Scheme S1) were purchased from Sigma-Aldrich (St. Louis, MO, USA). Maltose, maltohexaose, and maltoheptaose standards were purchased from Sigma-Aldrich (St. Louis, MO, USA). Maltotriose was purchased from Thermo Scientific (Ward Hill, MA, USA). Maltotetraose and maltopentaose were acquired from Biosynth Carbosynth (Louisville, KY, USA). GPC-grade dextran ladder was purchased from Sigma-Aldrich (Milwaukee, WI, USA). Lacto-*N*-difucohexaose I (LNDFH I) and lacto-*N*-difucohexaose II (LNDFH II) were obtained from Dextra Laboratories (Reading, UK). Bovine pancreas ribonuclease B (RNase B) and peptide-N-glycosidase F (PNGase F) were purchased from New England Biolabs (Ipswich, MA, USA). LC-MS grade ammonium formate was purchased from Sigma-Aldrich (St. Louis, MO, USA). All solvents that were used for the purification and analysis of the samples described herein were of HPLC-grade and purchased from Fisher Scientific (Nazareth, Pennsylvania, USA). The procedure for glycan release from RNase B is detailed in the Supporting Information.

### Synthesis of **Glyc**•RadiFluor

Briefly, 1,2-dimethyl-3,5-dinitrobenzene underwent a cyclization reaction with ethyl acrylate. The remaining methyl group was brominated followed by a TEMPO coupling reaction, hydrolysis of the ester into a carboxylic acid, conversion of the carboxylic acid into an acid chloride, the Schotten-Baumann reaction to form an amide, and the hydrogenation reaction to yield the final product. See Supporting Information for more details and for NMR spectra.

### Glycan Derivatization with **Glyc**•RadiFluor

A volume of 30 μL of 20 mM of the **Glyc•**RadiFluor tag in methanol and 20 μL of 1 mM of glycan in water were mixed together and evaporated *in vacuo*. The residue was redissolved in 50 mM sodium cyanoborohydride in anhydrous DMSO and glacial acetic acid (7:3 v/v) and the resulting mixture was incubated at 70 °C for 2 h. After completion of the glycan derivatization (Scheme S2), the mixture was evaporated *in vacuo* at 70 °C. For a total of three repetitions, the residue was vortexed and sonicated with 50 μL of HPLC-grade acetone to extract the unreacted tag and sodium cyanoborohydride, centrifuged for 5 min at 14 krpm, and the supernatant containing the free tag was recycled for future desalting and purification. After the final collection, the pellet containing the derivatized glycans was allowed to completely dry under ambient conditions for 10 min prior to LC-MS analysis.

### LC-FLR-HRMS/MS Analysis

A ThermoFisher Scientific Q Exactive Plus Oribtrap mass spectrometer coupled with a ThermoFisher Scientific Vanquish Flex UPLC instrument with a fluorescence detector was utilized for all the following experiments. The derivatized glycans were reconstituted in the initial state mobile phase consisting of 50 mM ammonium formate, pH 4.4 and LC-MS acetonitrile (14:86 v/v) and injections were made at 1.0 μL. An Xbridge Glycan BEH Amide *XP* column (2.5 μm particle size, L 150 mm x I.D. 3.0 mm) was utilized to separate **Glyc**•RadiFluor -derivatized glycan analytes according to the gradients described in the Supporting Information Table S1. The flow rate was set to 0.400 mL/min with a column temperature of 60.00 °C. The fluorescence detection took place with the parameters set to an excitation of 280.0 nm, an emission of 520.0 nm, detection sensitivity of 8 (arbitrary), and a scan rate of 5.00 Hz. The mass spectrometric experiments were conducted with the parameters set to a capillary voltage of +3.50 kV, a capillary temperature of 263 °C, sheath gas flow rate of 50 (arbitrary), auxiliary gas heater temperature of 425 °C, auxiliary gas flow rate of 13 (arbitrary), sweep gas flow rate of 3 (arbitrary), and an S-lens RF level of 50.0 (arbitrary). For the MS^2^ analysis of the analytes during each run, an isolation window of 4.0 *m*/*z* was implemented for each of the expected masses listed in the inclusion list at a resolution of 70,000. The HCD collisional energy was varied under normalized collision energy (NCE) mode.

## Results and Discussion

**Figure 2:**
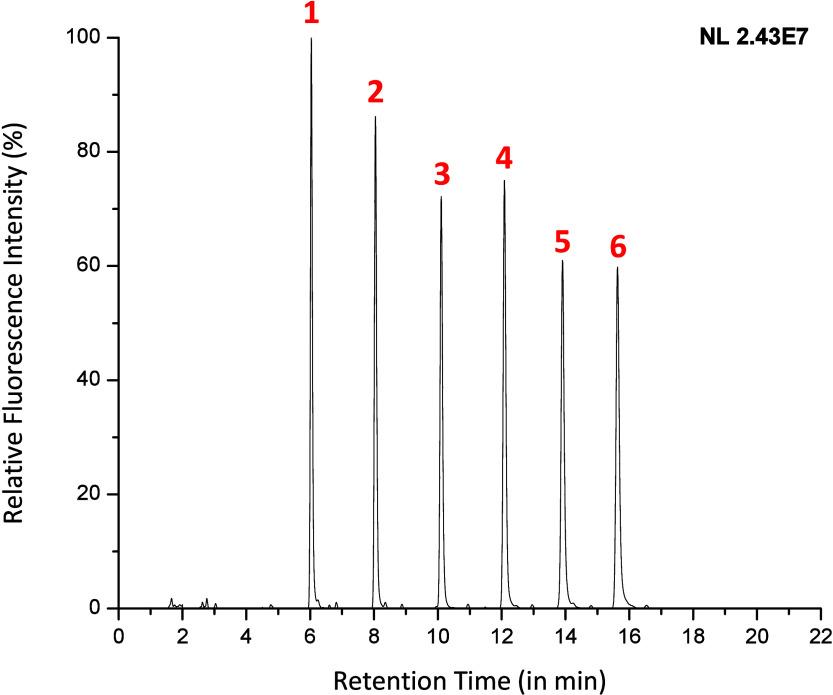
Fluorescence chromatogram depicting the separation for six **Glyc•**RadiFluor-derivatized glycans with variable glucose units that were prepared in an equimolar mixture (∼7.7 pmol per analyte); the peaks are identified as follows: (1) maltose, (2) maltotriose, (3) maltotetraose, (4) maltopentaose, (5) maltohexaose, and (6) maltoheptaose.

**Glyc**•RadiFluor, which enlists a free radical precursor, was synthesized in an effort to address challenges in modern glycan research by enhancing glycan characterization capabilities, facilitating sensitive fluorescence quantitation, enabling methylation and mass spectrometric ionization, and eliminating isomers at the glycan reducing end, with tolerable chemical stability. In particular, fluorescence detection possesses an advantage for glycan quantitation over other techniques because fluorescence responses are independent of the glycan structure, offering an invariable stoichiometry of “real” analytes regardless of analytical flaws such as matrix effects. The fluorescence detection capabilities of **Glyc•**RadiFluor were initially assessed by determining the optimal excitation and emission wavelengths at 280 ± 5 nm and 520 ± 5 nm, respectively. The correlation between the fluorescence intensity and the **Glyc•**RadiFluor concentration within the range of 1 nM and 1 μM were plotted and subjected to linear regression analysis (Supporting Information Figure S1). Compared to 2-AB, **Glyc•**RadiFluor is capable of producing a greater fluorescence response at nanomolar concentrations (i.e., **Glyc•**RadiFluor had at least a 6-fold greater response than 2-AB) signifying pronounced fluorescence sensitivity, enabling better detection and quantitative precision of glycans that naturally occur in low abundances (Supporting Information Figure S2). Upon determining and validating the optimal fluorescence wavelengths of **Glyc•**RadiFluor, a mixture of model glycans with progressively increasing number of glucose units (maltose, maltotriose, maltotetraose, maltopentaose, maltohexaose, and maltoheptaose) were derivatized via reductive amination. In brief, the importance of utilizing reductive amination for the derivatization protocol is to eliminate the interconversion between α-isomer, β-isomer, and open-chain isomer, which would otherwise complicate LC analysis by giving rise to additional chromatographic signals. After subsequent quantitative analysis, it was observed that the integration of the maltosaccharide peaks in Figure 2 returned values that were relatively equal, as shown in Supporting Information Table S2. Each analyte predominantly yielded a single peak that is associated with the open-chain isomer, rather than multiple peaks that correspond with the additional α and β isomers. This showcases the efficiency of the reducing agent to reduce the resulting Schiff base during the labeling reaction, thereby yielding a single isomer that will no longer interconvert at the glycan reducing end, establishing a simpler and more convenient analytical approach via LC for the detection of multiple analytes.

Next, the capability of **Glyc•**RadiFluor to characterize glycans was assessed by subjecting each of the six derivatized maltosaccharides to HCD. Upon HCD, the TEMPO free radical precursor fragments to generate a nascent, yet localized, free radical that simultaneously reacts with the glycan to induce cross-ring and glycosidic bond cleavages. Not only do the abundance of these fragmentations differ based on the structure or stereochemistry, but so do the type of fragmentations, providing valuable structural information for the differentiation and identification of a plethora of glycans and their isomers. All the product fragmentation ions described herein are assigned based on the Domon and Costello nomenclature for glycan fragmentations (Supporting Information Figure S3). Currently, no known nomenclature exists for the fragmentation ions corresponding to the reducing end open-chain isomer (saccharide unit 0). Therefore, a supplemental nomenclature system was accordingly devised to describe the results (Supporting Information Figure S4).

**Figure 3:**
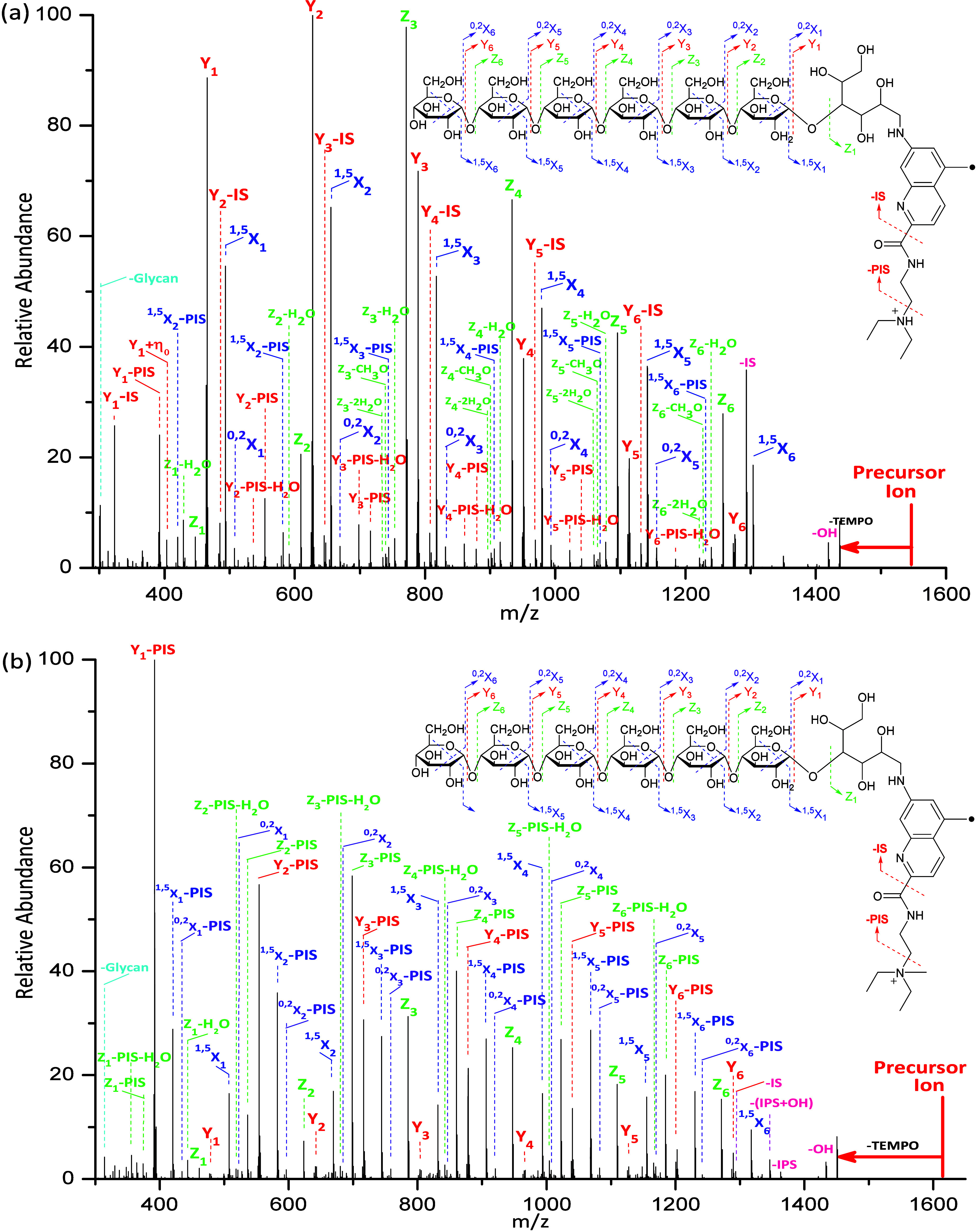
(a) The HCD spectrum with NCE of 30 (arbitrary) and fragmentation patterns for the singly protonated **Glyc•**RadiFluor-derivatized maltoheptaose and (b) the HCD spectrum with NCE of 30 (arbitrary) and fragmentation patterns for the methylated **Glyc•**RadiFluor-derivatized maltoheptaose. (PIS and IS are abbreviations for the partial ionization site and ionization site, respectively.)

As shown in Figure 3a, the singly protonated **Glyc•**RadiFluor-derivatized maltoheptaose was subjected to HCD normalized collisional energy of 30 (arbitrary) to fragment the TEMPO group and generate the free radical. The significance of the free radical has been reported in our previous report, wherein the glycosidic bond cleavage is dominant and very limited structural information is obtained without the free radical.^58^ With the combination of free radical chemistry and acid–base chemistry, simultaneous systematic cross-ring and glycosidic bond cleavages occur at each constituent saccharide unit, agreeing with our previous research studies.^59^ Among these fragmentations included the ^0,2^X- and ^1,5^X-type ions, which are cross-ring fragmentations, and the Y- and Z-type ions, which are glycosidic bond cleavages. Additionally, losses of H_2_O and CH_3_O were observed and are associated with the Z-type fragment ions. Interestingly, a systematic loss of the ionization site (IS) from Y ions, represented as Y–IS, are observed. The formation of the Y–IS ion is proposed to be initialized by the mobile proton as evidenced by the disappearance of the Y–IS ions upon HCD of methylated **Glyc**•RadiFluor-derivatized maltoheptaose wherein the mobile proton is replaced by a fixed charge. Meanwhile, ions of – (PIS+TEMPO), wherein PIS denotes the loss of the partial ionization site, – (IS+TEMPO), and – (OH+TEMPO) are observed. It is reported that although the fragmentations observed with the protonated species provide valuable information regarding the derivatized maltoheptaose, numerous fragmentations can be acid-catalyzed by a mobile charge and glycan rearrangements can occur.^58,59^ Such glycan rearrangements result in misleading fragmentations that deviate from the fragmentations that correspond to the actual structure. To eliminate glycan rearrangements and acid-catalyzed fragmentations, a fixed charge is introduced during sample preparation by reacting the **Glyc•**RadiFluor-derivatized maltoheptaose with iodomethane. The tertiary amine has great affinity for nucleophiles and is selectively attacked by the methyl cation to produce a positively charged quaternary amine. As shown in Figure 3b, the – IS and Y–IS ions are no longer observed, which demonstrates that the loss of IS is catalyzed by the mobile proton. The systematic ^0,2^X, ^1,5^X, Y, and Z ions are observed throughout the linear biopolymer. Interestingly, one more series of systematic ^0,2^X–PIS, ^1,5^X–PIS, Y–PIS, and Z–PIS ions are generated upon HCD. The ^0,2^X, ^1,5^X, Y, and Z ions are proposed to be generated via the loss of TEMPO, which simultaneously induces the formation of these ions. Meanwhile, the ^0,2^X–PIS, ^1,5^X–PIS, Y–PIS, and Z–PIS ions are proposed to be formed by being instantly induced via the loss of TEMPO+PIS.

**Figure 4:**
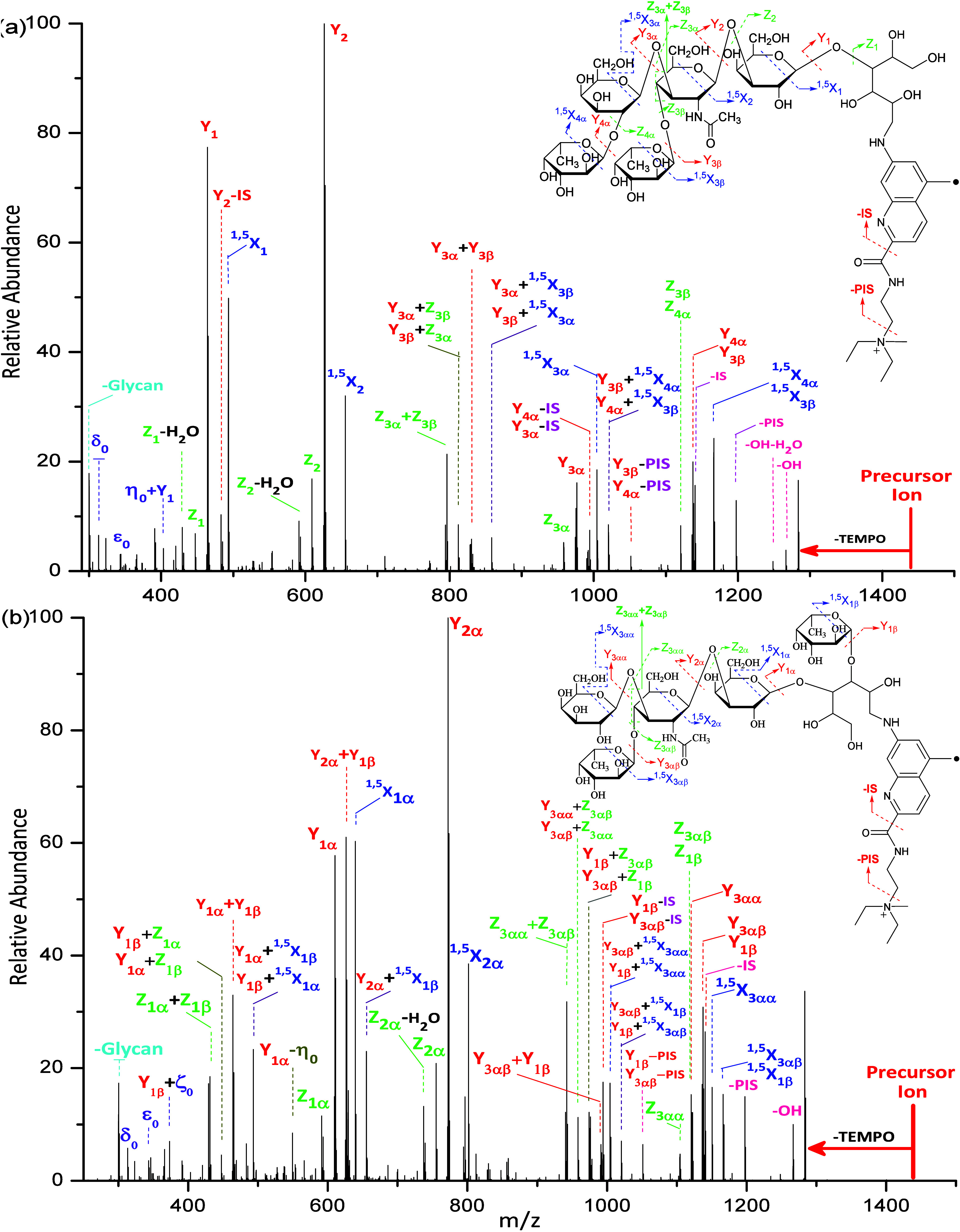
(a) The HCD spectrum with NCE of 28 (arbitrary) and fragmentation patterns for the singly protonated **Glyc•**RadiFluor-derivatized LNDFH I and (b) the HCD spectrum with NCE of 28 (arbitrary) and fragmentation patterns for the singly protonated **Glyc•**RadiFluor-derivatized LNDFH II. (PIS and IS are abbreviations for the partial ionization site and ionization site, respectively.)

**Figure 5:**
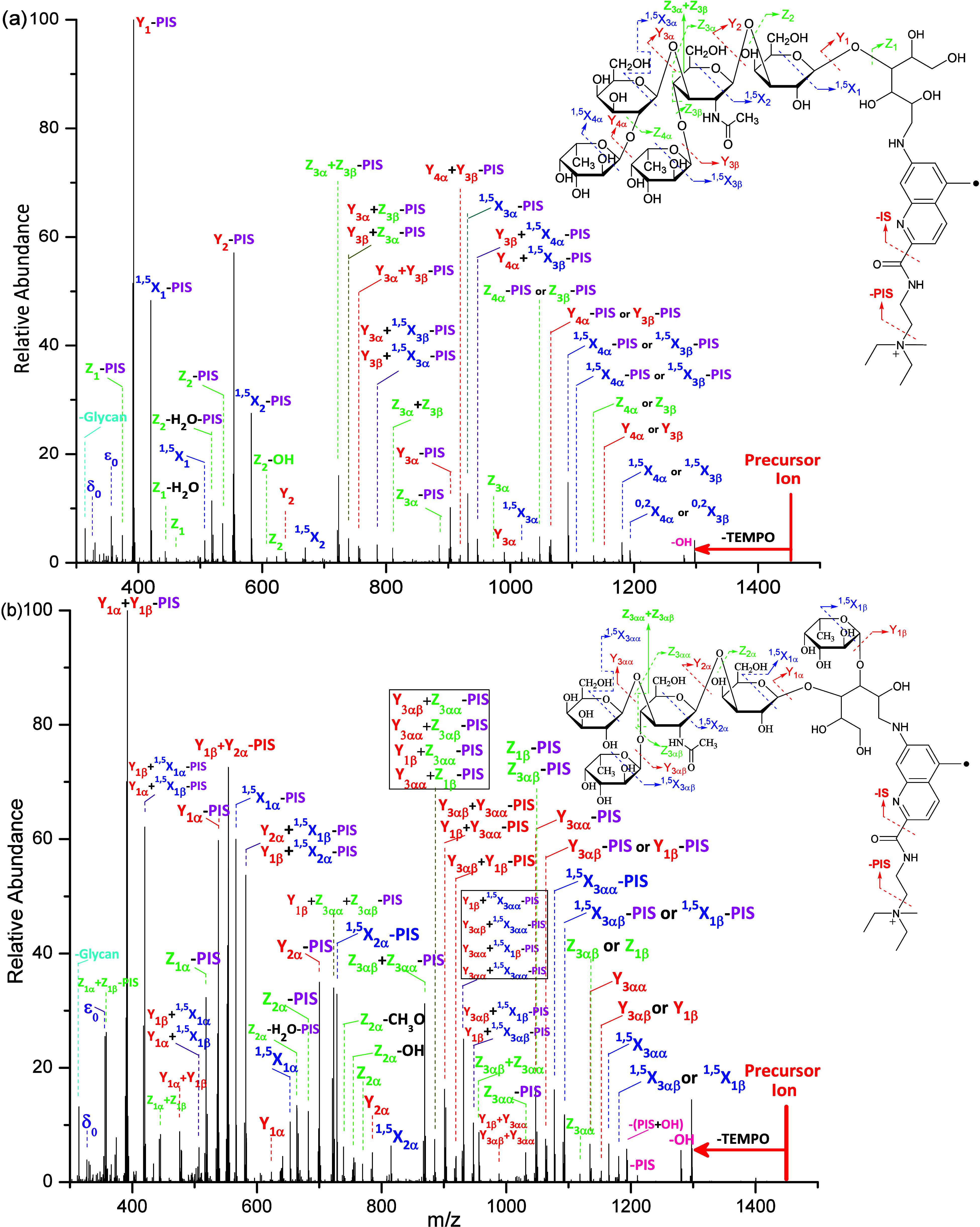
(a) The HCD spectrum with NCE of 32 (arbitrary) and fragmentation patterns for the methylated **Glyc•**RadiFluor-derivatized LNDFH I and (b) the HCD spectrum with NCE of 32 (arbitrary) and fragmentation patterns for the methylated **Glyc•**RadiFluor-derivatized LNDFH II. (PIS and IS are abbreviations for the partial ionization site and ionization site, respectively.).

The methodology involving **Glyc**•RadiFluor was further evaluated on a mixture of branched isobaric isomers. Lacto-*N*-difucohexaose I (LNDFH I) and lacto-*N*-difucohexaose II (LNDFH II) are hexasaccharide isomers that differ only in the linkage position of a single fucose subunit (structures shown in Figure 4). The fluorescence chromatogram depicts the hydrophilic liquid chromatography (HILIC) capability in separating the labeled LNDFH I and LNDFH II isomers (Supporting Information Figure S5). With the normalized HCD energy kept constant at 28 (arbitrary), the branched glycans generated more types of ions compared to linear maltoheptaose, including ^1,5^X, Y, Z, Y+^1,5^X, Y+Z, Z+Z, Y+Y, and Y-IS ions. According to our previous study, the Z+Z are unique ions at the branch sites, and the Y+^1,5^X and Y+Z ions are generated via combinational free radical chemistry and acid–base chemistry.^59^ The major differences in the HCD spectra for the branched isomers are the types of fragmentations and the relative abundances of the fragmentations (Figure 4). Although the fragmentation sites are similar in LNDFH I and LNDFH II, the HCD spectra for the protonated LNDFH II showed mass-shift fragmentations, notably the ^1,5^X_2α_, Y_2α_, and Z_2α_ product ions. This is particularly due to the branching nature of the glycan which, upon fragmentations occurring at saccharide unit 2 for LNDFH I (saccharide unit 2α for LNDFH II), results in a loss of four saccharide residues for LNDFH I but three saccharide residues for LNDFH II. This can be further exemplified by comparing the base peak pertaining to the Y_2α_ product ion for LNDFH II, which is at a higher mass-to-charge ratio than the base peak pertaining to the Y_2_ product ion for LNDFH I. Similar to maltoheptaose, HCD on methylated **Glyc•**RadiFluor-derivatized LNDFH I and LNDFH II generated systematic ^1,5^X, Y, Z, ^0,2^X–PIS, ^1,5^X–PIS, Y–PIS, and Z–PIS ions. Moreover, Z+Z, Z+Z–PIS, Y+Z–PIS, and Y+Y–PIS ions are generated providing more structural information and allowing the differentiation of LNDFH I and LNDFH II (Figure 5).

To normalize the retention time of the glycans, a dextran ladder standard solution was prepared and derivatized with **Glyc**•RadiFluor. A dextran ladder standard is often used in glycan analysis to define the retention time of glycans in terms of glucose units. As shown in Figure S6a, the dextran ladder was separated and detected via fluorescence and each peak was defined in terms of the number of glucose units (GUs). Based on the data obtained by the derivatized dextran ladder, the logarithm of GUs versus retention time is plotted to construct a curve (Figure S6b). This enables arithmetic conversions of the retention time of all glycan analytes into a measure of GUs. By describing glycan analytes in terms of GUs, the observed retention time from one method can be cross-referenced with those acquired through other methods. Because the retention times of glycan analytes are relative to the retention times of the dextran ladder for a given method, any glycan can be assigned with a unique GU literature value. The GU values can be a supplementary glycan characterization technique while free radical mediated characterization via MS remains a more intricate and robust technique toward glycan characterization, especially for samples involving unknown glycans.

**Figure 6:**
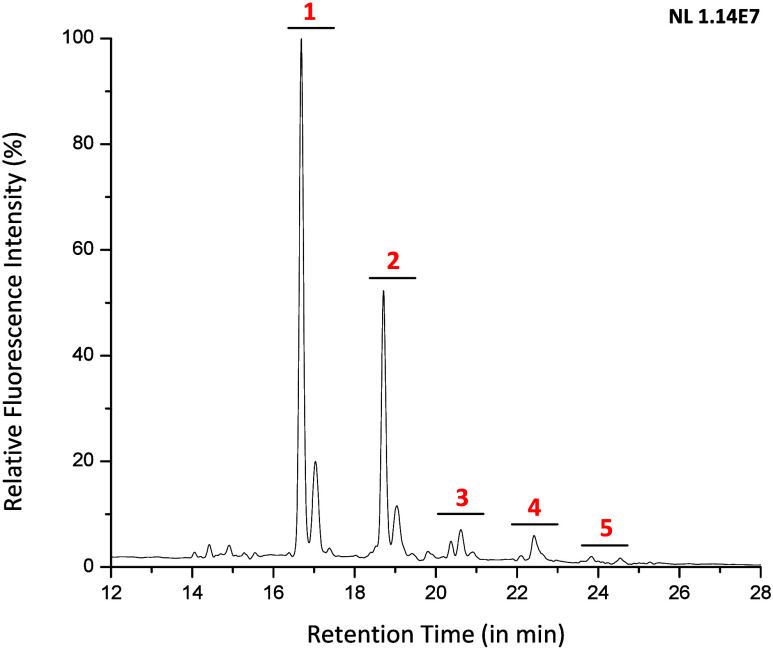
Fluorescence chromatogram depicting the separation of **Glyc•**RadiFluor-derivatized RNase B high-mannose *N*-glycans; the peaks are identified as follows: (1) Man_5_(GlcNAc)_2_, (2) Man_6_(GlcNAc)_2_, (3) Man_7_(GlcNAc)_2_, (4) Man_8_(GlcNAc)_2_, and (5) Man_9_(GlcNAc)_2_.

Analysis of *N*-glycans from ribonuclease B (RNase B) was performed where they were first enzymatically released from the asparagine residues by peptide-*N*-glycosidase F (PNGase F). RNase B is typically employed as a positive control for endoglycosidases and is a high mannose glycoprotein decorated with *N*-glycans of the architecture Man_5–9_(GlcNAc)_2_. After enzymatic release, the high mannose *N*-glycans were derivatized with **Glyc**•RadiFluor through reductive amination to eliminate reducing end isomers. As portrayed in the fluorescence chromatogram of derivatized *N*-glycans in Figure 6, Man_5_(GlcNAc)_2_ exhibited the most pronounced abundance followed by Man_6_(GlcNAc)_2_, and last, Man_7–9_(GlcNAc)_2_ with far lower abundances. In the HCD mass spectrum belonging to Man_5_(GlcNAc)_2_, the fragmentations provide key information to determine the isomeric structure. For instance, the intensity of the loss of two mannose subunits and four mannose subunits resulting from the mobile proton as previously reported is relatively lower than the loss of one mannose, three mannose, and five mannose subunits, which provides further evidence of the structure of the glycan (Figure S7a).^76^ Since the ^1,5^X_4α’/4α’’/3β_+Y_4α’/4α’’/3β_ product ions associated with the loss of two mannose subunits has six total possible fragmentation pathways, the probability of yielding such a loss increases relative to the two possible fragmentation pathways for the ^1,5^X_3α_+Y_3β_ or ^1,5^X_3β_+Y_3α_ product ions associated with the loss of four mannose subunits. For fragmentations belonging to the methylated glycan in Figure S7b, the only possible pathways leading to the loss of two mannose subunits (and similarly for the loss of four mannose subunits) occurs after the partial fragmentation of the ionization site, which is proposed to yield a mobile proton (Scheme S3). This observation provides additional evidence that supports the isomeric structure and branching locations of the mannose subunits for Man_5_(GlcNAc)_2_, as shown in Figure S7. Likewise, the HCD mass spectra for Man_6_(GlcNAc)_2_ provides evidence that the predominant isomer present on RNase B is biantennary with a single saccharide elongation occurring at the 3β unit from the Man_5_(GlcNAc)_2_ predecessor (Figure S7). In its protonated form (Figure S7a), HCD of Man_6_(GlcNAc)_2_ results in fragmentations of high abundance and are associated with the loss of one, two, three, and six mannose residues, but not four and five mannose residues. Similarly, the methylated and derivatized Man_6_(GlcNAc)_2_ yields product ions comparable to the protonated form, with some occurring with the additional loss of the PIS (Figure S8b). The proposed isomeric structure of Man_6_(GlcNAc)_2_ therefore satisfies the fragmentation data, and vice versa.

## Conclusion

In this text, we have described a robust and modern glycan analytical approach employing a novel fluorescent free radical tag, **Glyc•**RadiFluor, which is capable of both quantitation and characterization of glycans. This novel tag possesses four essential chemical sites: (1) the quinolinyl fluorophore serves as a quantitation functionality that facilitates high sensitivity and glycan structure-independent fluorescence; (2) the free radical precursor generates a well-defined nascent free radical upon collisional activation, which further and simultaneously induces systematic, predictable, and efficient fragmentations for glycan characterization; (3) the amino group selectively derivatizes glycans at unique reducing termini via reductive amination and thereby eliminates the interconversion between multiple isomers at the reducing end; and (4) the tertiary amine functions as the highly basic site for the formation and retainment of a mobile or fixed charge, significantly enhancing the sensitivity of positive electrospray ionization mass spectrometry (ESI/MS). We have demonstrated the robustness of this tag toward glycan quantitation and characterization using various glycan samples including standardized linear maltosaccharides, branched isobaric hexasaccharides, and enzymatically released *N*-glycans from RNase B. Given the potential of this tag in enhancing glycan analysis, future studies will probe into the application of this novel tag to studying *N*-glycans extracted from tissue under normal versus aberrant conditions and developing strategies to address the shortcomings behind studying *O*-glycans. Lastly, future research efforts will investigate a subtle variation of this tag, where the ionization site functionality is replaced with an acidic group, to address glycan analysis in the negative ion mode.

## Supporting Information Available

Gradient for the HILIC-FLD-ESI+ analysis of the labeled glycans, HILIC-FLD-ESI+ analysis of the labeled maltosaccharides, intensity of fluorescence emitted by **Glyc•**RadiFluor, comparison of the fluorescence of **Glyc•**RadiFluor and 2-AB, nomenclature for glycan fragmentation ions, HILIC spectra and fluorescence detection of LNDFH I and II, fluorescence of the neutral **Glyc•**RadiFluor-derivatized dextran ladder, HCD spectra, overview of the synthesis and glycan derivatization, proposed mechanism for fragmentation of the quaternary amine, synthesis procedures, and NMR data of **Glyc•**RadiFluor and its intermediates (PDF)
