# Supplementary material for: Sensitive Fluorescence Quantitation and Efficient Free Radical Characterization of N-Glycans via LC-FLR-HRMS/MS with a Novel Fluorescent Free Radical Tag
Source: Anal Chem. 2025 Mar 25;97(13):7118–27. doi: 10.1021/acs.analchem.4c06294 (PMC11983377; doi:10.1021/acs.analchem.4c06294)
Supplement: Supplementary file 1 — ac4c06294_si_001.pdf [file ac4c06294_si_001.pdf]

Supplementary Information

Sensitive Fluorescence Quantitation and Efficient Free Radical Characterization of *N*-Glycans via LC-FLR-HRMS/MS with a Fluorescent Free Radical Tag

Rayan Murtada, CJ Szafranski, Maria Tevletidis, Shane Finn, Wilthon Gilles, Tabia Tahsin, and Jinshan Gao*

Department of Chemistry and Biochemistry, Montclair State University, 1 Normal Avenue, Montclair, New Jersey, 07043, United States
Sokol Institute of Pharmaceutical Life Sciences, Montclair, New Jersey, 07043, United States

1. Rayan Murtada and CJ Szafranski have made equal contributions to this work.

Table of Contents

Table S1. The gradient for the HILIC-FLD-ESI+ MS analysis of the labeled glycans.
Table S2. HILIC-FLD-ESI+ MS analysis of the labeled maltosaccharides.
Figure S1. The intensity of fluorescence emitted by the fluorescent tag **Glyc**•RadiFluor.
Figure S2. Fluorescence comparison of **Glyc**•RadiFluor and 2-AB.
Figure S3. The nomenclature for the glycan fragmentation ions.
Figure S4. The novel nomenclature for the reducing end open-chain isomer fragmentation ions.
Figure S5. The HILIC separation and fluorescence detection of **Glyc**•RadiFluor derivatized LNDFH I and II.
Figure S6. Fluorescence of the neutral **Glyc•**RadiFluor-derivatized dextran ladder.
Figure S7. The HCD spectrum for the protonated and methylated **Glyc•**RadiFluor-derivatized $Man_5(GlcNAc)_2$.
Figure S8. The HCD spectrum for the protonated and methylated **Glyc**•RadiFluor-derivatized Man6(GlcNAc).
Scheme S1. Overview of the synthesis of the novel fluorescent and free radical tag.
Scheme S2. Glycan derivatization at the reducing end with the **Glyc•**RadiFluor tag.
Scheme S3. Proposed mechanism for the fragmentation of the quaternary amine.
Synthesis procedure and NMR data of Glyc•RadiFluor and intermediates.

Mobile Phase A: 50 mM ammonium formate, pH 4.4
Mobile Phase B: LC-MS acetonitrile

Time	Flow Rate (mL/min)	%A	%B	Curve
0	0.4	14	86	4
35.0	0.4	50	50	4
36.5	0.2	100	0	6
39.5	0.2	100	0	5
43.1	0.2	14	86	6
47.6	0.4	14	86	5
55.0	0.4	14	86	5

Table S1. The gradient for the HILIC-FLD-ESI+ MS analysis of the labeled glycans.

Peak	Analyte	Peak Area	Theoretical Quantity (pmol)	Peak Mass $[M+H]^+$	Theoretical Mass $[M+H]^+$	Mass Error (ppm)
1	Maltose	1.090e8	7.8	782.4545	782.45460	-0.09
2	Maltotriose	1.044e8	7.5	944.5075	944.50742	0.09
3	Maltotetraose	1.026e8	7.4	1106.5604	1106.56025	0.16
4	Maltopentaose	1.096e8	7.9	1268.6141	1268.61307	0.84
5	Maltohexaose	1.050e8	7.6	1430.6661	1430.66589	0.17
6	Maltoheptaose	1.091e8	7.8	1592.7189	1592.71872	0.10

Table S2. The peak integrations and peak masses for the HILIC-FLD-ESI+ MS analysis of the labeled maltosaccharides.

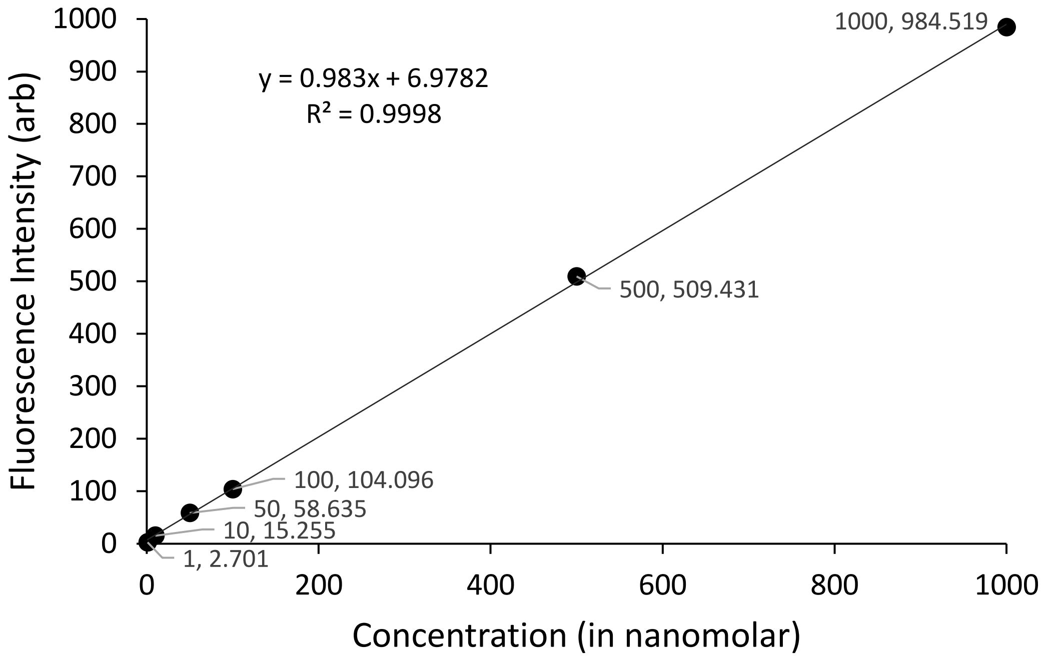

Figure S1. The intensity of fluorescence emitted by the fluorescent tag **Glyc**•RadiFluor at concentrations between 1 nM and 1 µM; λ_{ex} = 280 nm and λ_{em} = 520 nm.

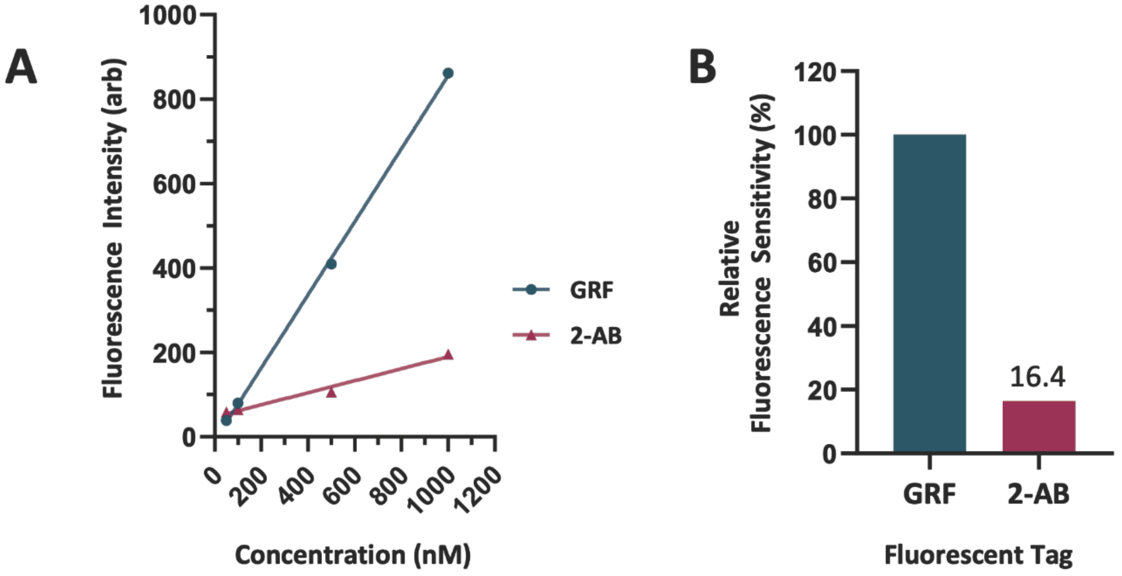

Figure S2. **(A)** Fluorescence intensity emitted by fluorescent tags **Glyc**•RadiFluor (GRF; λ_{ex} = 280 nm and λ_{em} = 520 nm) and 2-aminobenzamide (2-AB; λ_{ex} = 330 nm and λ_{em} = 420 nm); PMT detector voltage 760 V. **(B)** Fluorescence sensitivity of 2-AB relative to GRF as measured by the slope values computed via linear regression analysis. (R^2 > 0.98 for all data.)

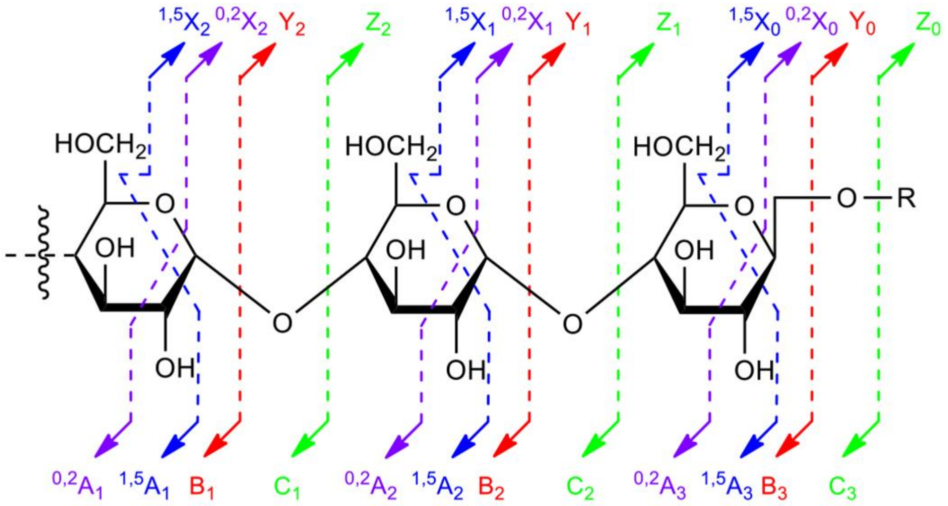

Figure S3. The nomenclature for the glycan fragmentation ions.

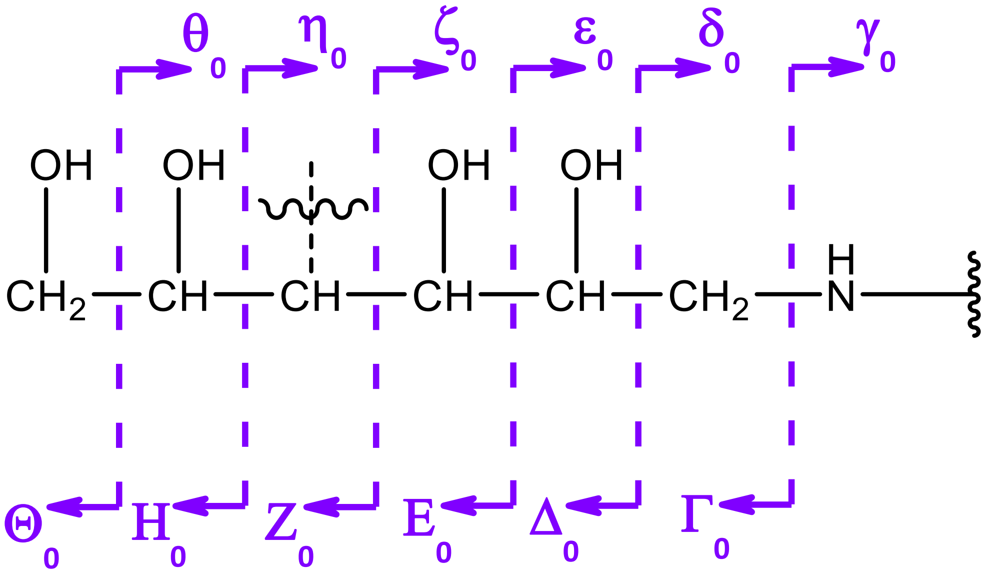

Figure S4. The novel nomenclature for the reducing end open-chain isomer fragmentation ions.

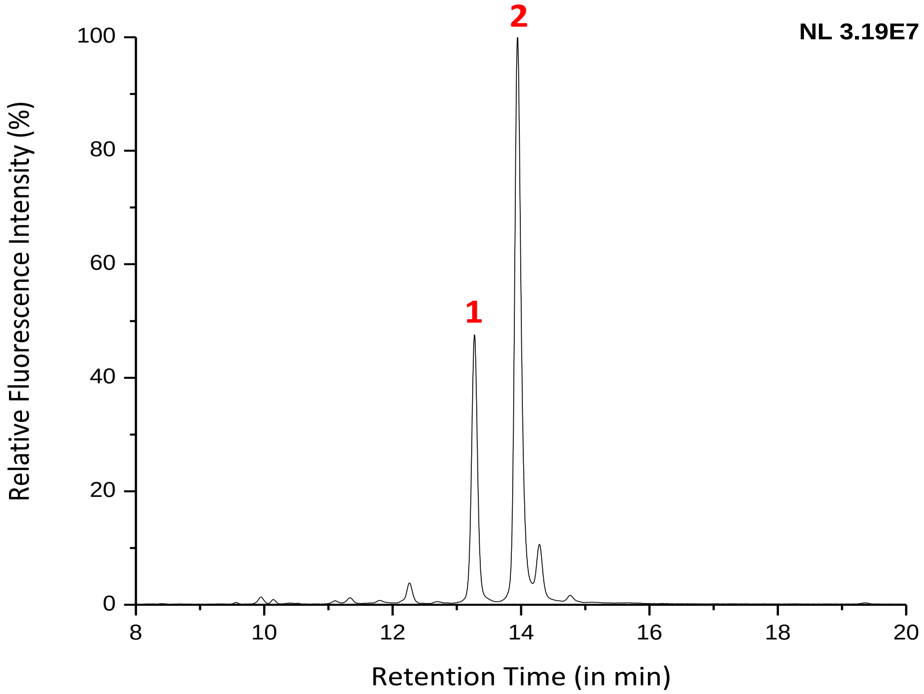

Figure S5. The HILIC separation and fluorescence detection of a sample containing unknown quantities of the branched isobaric glycans lacto-*N*-difucohexaose I (LNDFH I; peak 2) and lacto-*N*-difucohexaose II (LNDFH II; peak 1) after labeling with **Glyc**•RadiFluor.

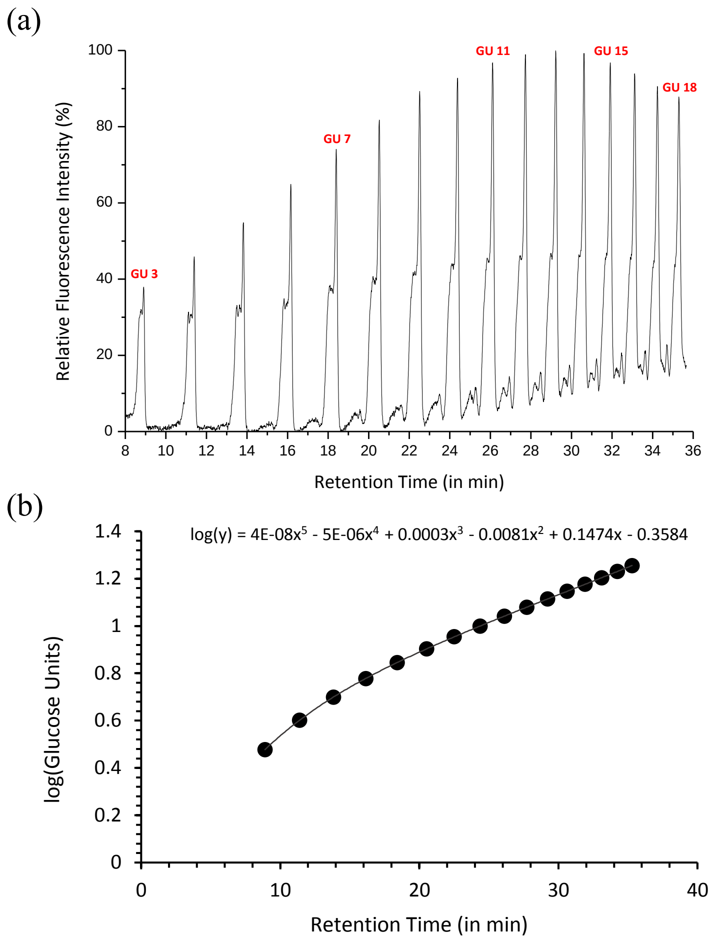

Figure S6. (**a**) A fluorescence chromatogram of the neutral **Glyc•**RadiFluor-derivatized dextran ladder separated via hydrophilic interaction liquid chromatography (HILIC). (**b**) A logarithmic plot of the glucose unit value versus the retention time for the neutral **Glyc•**RadiFluor-derivatized dextran ladder that is specific for HILIC-FLD analysis.

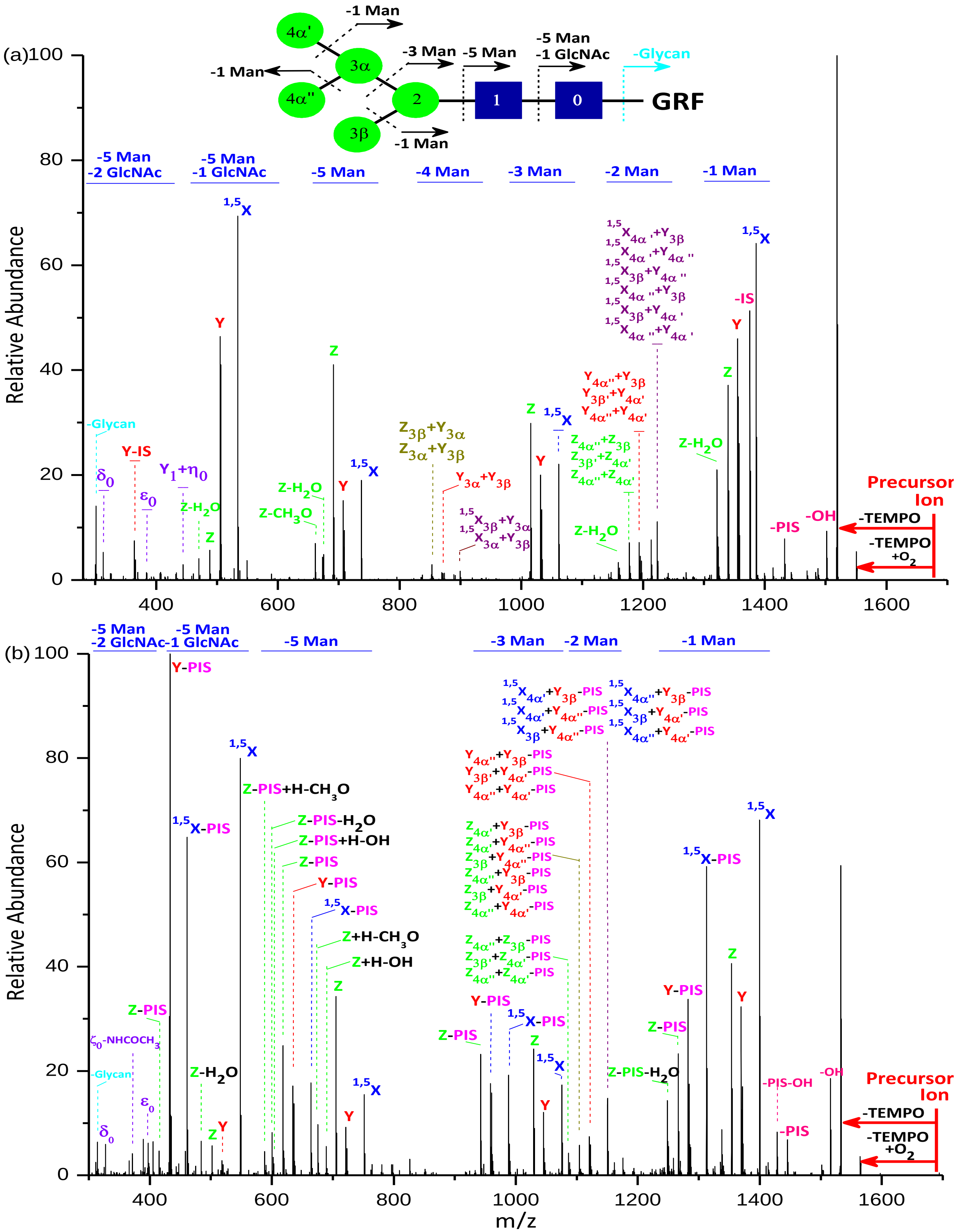

Figure S7. (a) The HCD spectrum with NCE of 25 (arbitrary) for the protonated **Glyc•**RadiFluor-derivatized $Man_5(GlcNAc)_2$, and **(b)** the HCD spectrum with NCE of 28 (arbitrary) for the methylated **Glyc•**RadiFluor-derivatized $Man_5(GlcNAc)_2$. (PIS and IS are abbreviations for the partial ionization site and ionization site, respectively.)

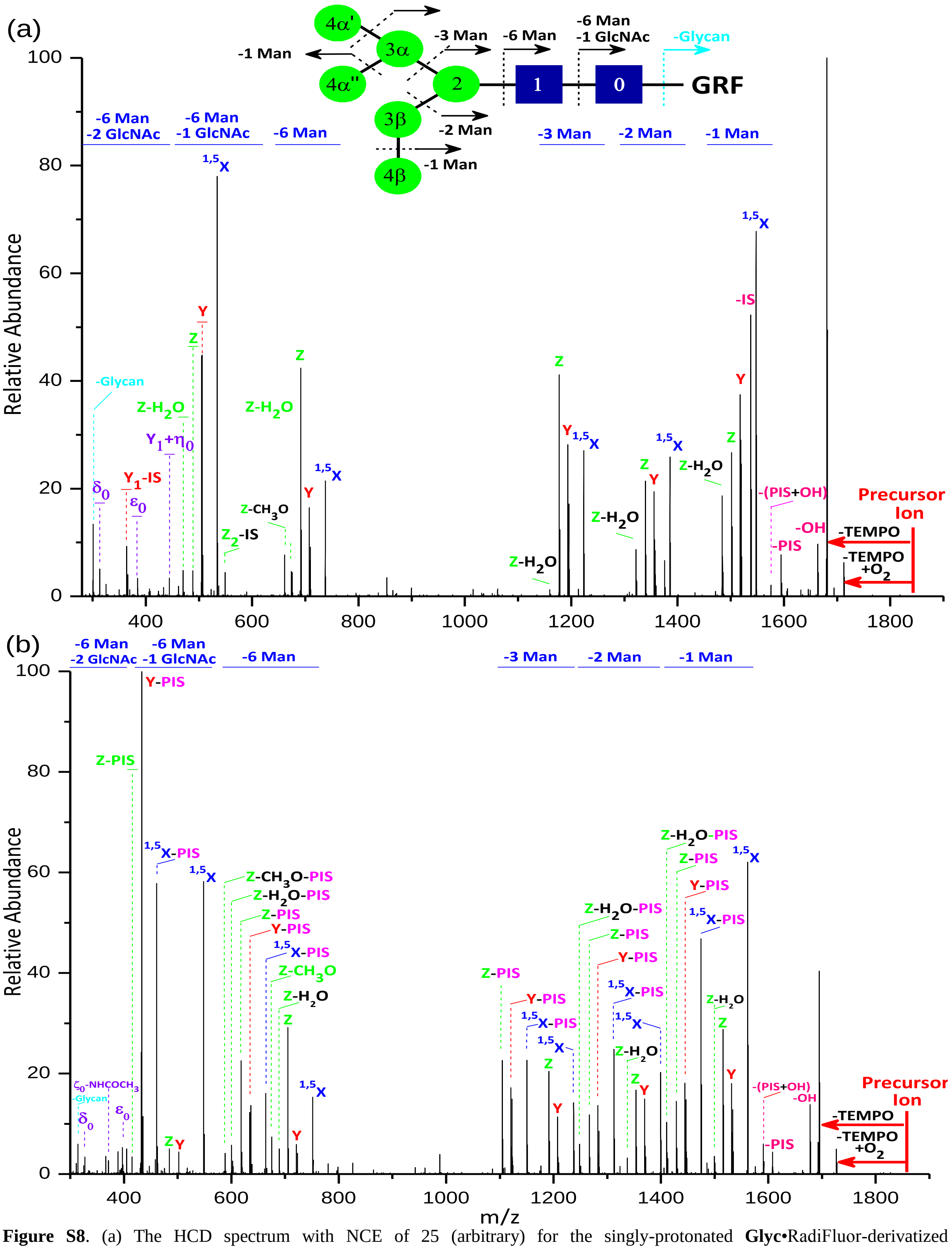

Figure S8. (a) The HCD spectrum with NCE of 25 (arbitrary) for the singly-protonated **Glyc**•RadiFluor-derivatized Man6(GlcNAc)2, and (b) the HCD spectrum with NCE of 28 (arbitrary) for the singly-methylated **Glyc**•RadiFluor-derivatized Man6(GlcNAc)2. (PIS and IS are abbreviations for the partial ionization site and ionization site, respectively.)

***N*-Glycan Deglycosylation of RNase B.** The manufacturer's protocol was followed for the deglycosylation of *N*-glycans from RNase B. To ensure full deglycosylation of the glycoprotein, the incubation step was elongated to 14 h. The resulting aqueous solution was cooled down to room temperature and subjected to purification by porous graphitic carbon solid-phase extraction. The cartridge was activated with acetonitrile and equilibrated with 5% acetonitrile prior to sample application. Afterwards, the cartridge was washed with 1 mL of water for a total of five times and the glycans were eluted and collected through a 0.2 µm nylon filter with 250 µL of 40% ACN with 0.1% formic acid for a total of four times. The eluate was evaporated *in vacuo* at 60° C prior to following the derivatization step.

Fluorescence Assays. Variable concentrations were prepared to evaluate the fluorescence responses of the **Glyc•**RadiFluor and 2-aminobenzamide tags. For the **Glyc•**RadiFluor tag, an excitation scan with the emission mode set to zero order was initially performed via the Agilence Cary Eclipse fluorescence spectrophotometer on a 100 µM sample to determine the preliminary excitation wavelength values. Each excitation wavelength was then subjected to an emission scan to determine the wavelength pair with the optimal fluorescence intensity. With the parameters set to a +700 V PMT detector voltage, 5 nm excitation and emission slits, and 1.000 s averaging time, the optimal **Glyc•**RadiFluor tag excitation and emission wavelengths at 280 nm and 520 nm, respectively, were selected for the construction of a concentration-dependent fluorescent plot.

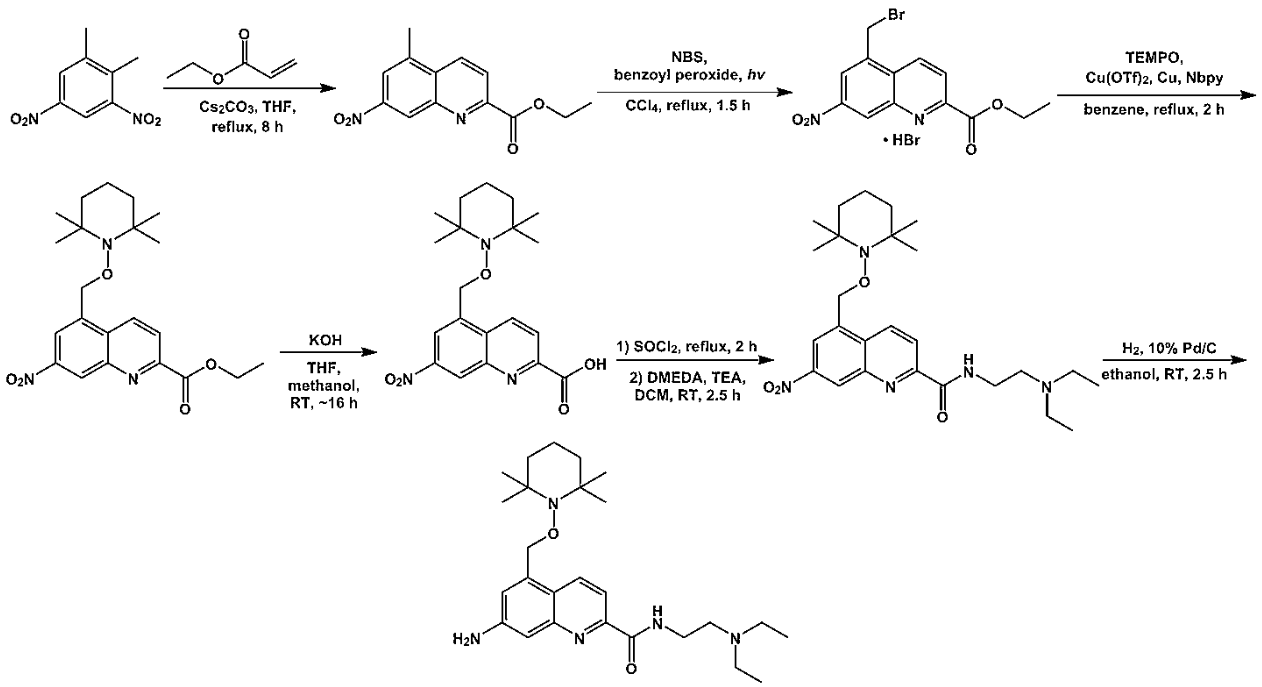

Scheme S1. Overview of the synthesis of the novel fluorescent and free radical tag.

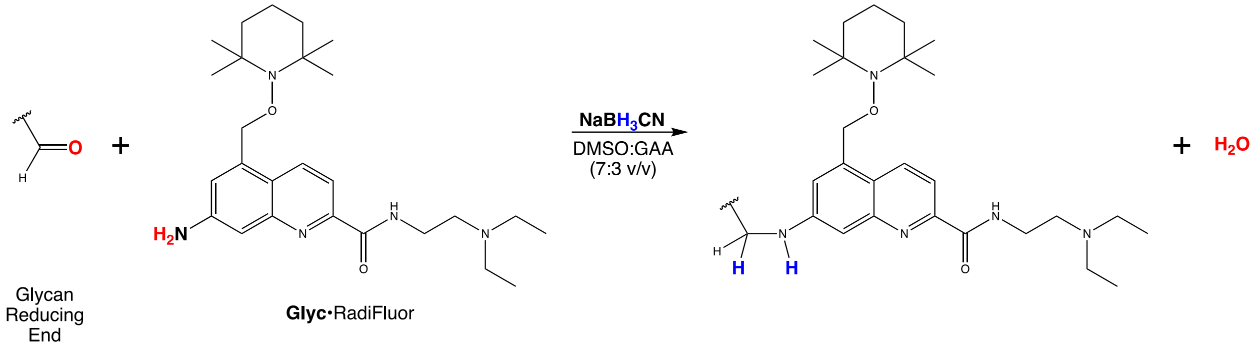

Scheme S2. Glycan derivatization at the reducing end with the **Glyc•**RadiFluor tag.

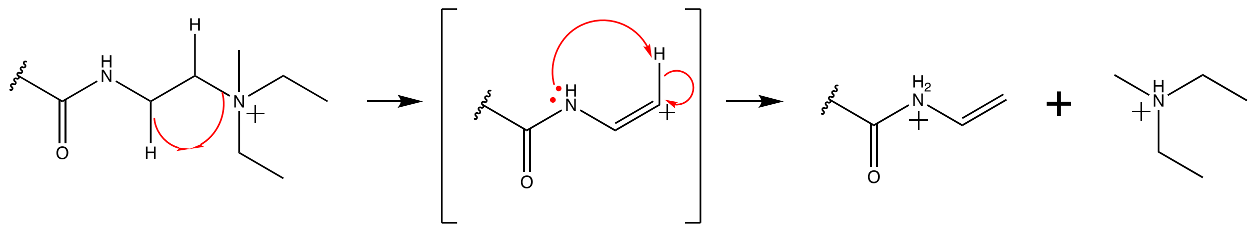

Scheme S3. Proposed mechanism for the fragmentation of the quaternary amine, thereby yielding a mobile charge.

Synthesis of Glyc•RadiFluor

As shown in **Scheme S1**, the synthesis of the **Glyc•**RadiFluor tag begins with a cyclization reaction involving a substituted benzene and an olefin. The final product was obtained through a series of six reactions, labeled **I-VI**.

***Ethyl 5-methyl-7-nitro-2-quinolinecarboxylate* (I)**

In a clean, oven-baked flask equipped with a stir bar, 1,2-dimethyl-3,5-dinitrobenzene (5 mmol) was allowed to react with ethyl acrylate (2 equiv.) in a solution of cesium carbonate (3 equiv.) in anhydrous tetrahydrofuran (25 mL) under argon. After 8 h of reflux, the crude mixture was allowed to cool down and was evaporated *in vacuo* before the product was extracted from the residue with dichloromethane. The collections were purified via silica gel flash chromatography by using ethyl acetate and hexanes. The desired product was obtained as a yellow solid (360.5 mg, 28% yield). ^{1}H NMR (400 MHz, $CDCl_3$, δ): 9.10 (1H, s), 8.55 (1H, d), 8.37 (1H, d), 8.27 (1H, s), 4.63-4.58 (2H, q), 2.85 (3H, s), 1.53 (3H, t); ^{13}C NMR (100 MHz, $CDCl_3$, δ): 164.65, 150.06, 148.10, 146.89, 137.31, 133.96, 131.59, 124.99, 123.39, 121.76, 62.67, 18.99, 14.32. MS ESI calcd. for $C_{13}H_{13}N_2O_4^+$ ($[M+H]^+$): 261.09; found 261.25.

***Ethyl 5-bromomethyl-7-nitro-2-quinolinecarboxylate hydrobromide* (II)**

A flame-dried flask was charged with ethyl 5-methyl-7-nitro-2-quinolinecarboxylate (1.385 mmol), *N*-bromosuccinimide (1.25 equiv.), benzoyl peroxide (0.1 equiv.), and vacuumed for 30 min before placing them under an atmosphere of argon. The reactants were dissolved in anhydrous carbon tetrachloride (70 mL) and refluxed for 1.5 h with a light catalyst (250 W) and stirring. Upon completion, the crude mixture was allowed to cool down to room temperature and was purified via silica gel flash chromatography with ethyl acetate and hexanes (3:7). The desired product was converted to a salt by adding hydrobromic acid followed by 30 min of stirring and completely

evaporating the solvents under reduced pressure. MS ESI calcd. For $C_{13}H_{12}{}^{79}BrN_2O_4^+$ ([M+H]$^+$): 339.00; found 339.17.

Ethyl 5-(TEMPO)methyl-7-nitro-2-quinolinecarboxylate **(III)**

The ethyl 5-bromomethyl-7-nitro-2-quinolinecarboxylate hydrobromide (assuming 1.385 mmol) from the previous step was converted back into neutral form by dissolving the salt in water and basifying the solution with sodium hydroxide until pH ~7. The organic compound was extracted with benzene (60 mL) and the collections were dried over anhydrous sodium sulfate. The dried benzene layer was transferred to a flame-dried flask and degassed with argon for 30 min after dissolving TEMPO (1.2 equiv.), $Cu(Otf)_2$ (0.1 equiv.), Nbpy (0.4 equiv.), and copper powder (1.2 equiv.). With stirring, the solution was refluxed for 2 h under argon. After cooling down to room temperature, the crude mixture was filtered through a short pad of silica gel and eluted with ethyl acetate. The organic layer was transferred to a separatory funnel and washed with saturated NH_4Cl, 1 M NH_4OH, and brine. The organic layer was dried over anhydrous sodium sulfate before purification via silica gel flash chromatography with ethyl acetate and hexanes (1:5). The desired product was obtained as a white to off-white solid (144.8 mg, 25% overall yield for bromination followed by TEMPO coupling). ^{1}H NMR (400 MHz, $CDCl_3$, δ): 9.18 (1H, s), 8.58 (1H, d), 8.50 (1H, s), 8.36 (1H, d), 5.36 (2H, s), 4.63-4.58 (2H, q), 1.60-1.11 (21H, m, TEMPO+CH_3); ^{13}C NMR (100 MHz, $CDCl_3$, δ): 164.59, 150.11, 148.26, 146.86, 137.59, 133.92, 130.25, 126.06, 123.41, 120.16, 75.68, 62.70, 60.25, 39.68, 38.11, 33.36, 29.69, 20.24, 14.64. MS ESI calcd. for $C_{22}H_{30}N_3O_5^+$ ([M+H]$^+$): 416.22; found 416.42.

***Ethyl 5-(TEMPO)methyl-7-nitro-2-quinolinecarboxyic acid* (IV)**

Ethyl 5-(TEMPO)methyl-7-nitro-2-quinolinecarboxylate (0.3485 mmol) was added to a clean flask and dissolved with THF (10 mL) and methanol (10 mL). An aqueous solution of 2 M KOH (20 mL) was slowly added to the mixture. The reaction was allowed to continue with stirring at room temperature. Upon completion, the crude mixture was evaporated under reduced pressure to remove THF and methanol. An aqueous solution of 2 M HCl was added to the crude mixture until pH ~7 before extracting with ethyl acetate five times and drying the organic layer over anhydrous sodium sulfate. The organic layer was then purified via silica gel flash chromatography with methanol and DCM (1:20). The desired product was obtained as a white solid (98.7 mg, 73% yield). ^{1}H NMR (400 MHz, methanol-*d4,* δ): 8.99 (1H, s), 8.73 (1H, d), 8.52 (1H, s), 8.39 (1H, s), 5.42 (2H, s), 1.67-1.22 (18H, m, TEMPO); ^{13}C NMR (100 MHz, methanol-*d4*, δ): 173.17, 167.23, 151.83, 149.77, 147.75, 139.57, 136.02, 131.44, 129.44, 126.06, 120.77, 76.78, 61.51, 40.91, 33.87, 23.84, 21.01, 18.13, 14.51. MS ESI calcd. for $C_{20}H_{26}N_3O_5^+$ ($[M+H]^+$): 388.19; found 388.50.

***N-[2-(Diethylamino)ethyl]-5-(TEMPO)methyl-7-nitro-2-quinolinecarboxamide* (V)**

To an oven-baked flask, ethyl 5-(TEMPO)methyl-7-nitro-2-quinolinecarboxyic acid (0.2543 mmol) was added and vacuumed for 30 min before being dissolved in thionyl chloride (10 mL). The reaction was allowed to occur by refluxing with stirring for 2 h. Afterwards, the thionyl chloride was evaporated *in vacuo* and the residue was immediately redissolved in anhydrous DCM (5 mL). In a separate oven-baked flask, *N*,*N*-diethylethylenediamine (50 µL) and anhydrous trimethylamine (140 µL) were dissolved in anhydrous DCM (5 mL). The acid chloride was added to this mixture in a dropwise fashion over the course of 10 min. After 2.5 h of stirring at room temperature, the mixture was diluted with 15 mL of DCM and washed with saturated aqueous sodium bicarbonate once. The aqueous layer was then extracted with DCM twice and the combined

organic layers were dried over anhydrous sodium sulfate. The organic layer was then subjected to purification via silica gel flash chromatography with methanol and DCM. The desired product was obtained as a white solid (100.8 mg, 82% yield). ^{1}H NMR (400 MHz, $CDCl_3$, δ): 8.92 (1H, s), 8.63-8.47 (4H, m), 5.35 (2H, s), 3.60 (2H, q), 2.77-2.63 (6H, m), 1.62-1.11 (24H, m, TEMPO+2CH_3); ^{13}C NMR (100 MHz, $CDCl_3$, δ): 163.41, 151.87, 148.09, 145.77, 137.75, 133.80, 130.12, 125.17, 121.50, 119.33, 75.59 (overlap with solvent peak), 60.20, 51.48, 47.14, 39.66, 37.45, 33.30, 20.19, 11.94. MS ESI calcd. for $C_{26}H_{40}N_5O_4^+$ ([M+H]$^+$): 486.31; found 486.50.

7-Amino- N-[2-(diethylamino)ethyl]-5-(TEMPO)methyl -2-quinolinecarboxamide **(VI)**

N-[2-(Diethylamino)ethyl]-5-(TEMPO)methyl-7-nitro-2-quinolinecarboxamide (0.2076 mmol) was added to a clean flask equipped with a stir bar. The solid was dissolved in absolute ethanol (5 mL) prior to an addition of 10% Pd/C (0.5 equiv.). A source of hydrogen gas was attached to the flask and the reaction was allowed to occur at room temperature with stirring for 2.5 h. Upon completion, the crude mixture was filtered through sand and celite followed by elution with ethanol. The organic layer was purified via silica gel flash chromatography with methanol and DCM to obtain the final pure product as a yellow solid (51.5 mg, 54% yield). ^{1}H NMR (400 MHz, $CDCl_3$, δ): 8.59 (1H, t), 8.26 (1H, d), 8.02 (1H, d), 7.17 (1H, s), 7.12 (1H, s), 5.21 (2H, s), 4.16 (2H, s), 3.57 (2H, q), 2.78-2.64 (6H, m), 1.58-1.109 (24H, m, TEMPO+2CH_3); ^{13}C NMR (100 MHz, $CDCl_3$, δ): 164.94, 149.71, 148.80, 147.54, 136.21, 133.19, 121.48, 118.62, 115.08, 108.27, 75.91 (overlap with solvent peak), 60.06, 51.71, 47.15, 39.68, 37.23, 33.00, 20.25, 11.73. MS ESI calcd. for $C_{26}H_{42}N_5O_2^+$ ([M+H]$^+$): 456.33; found 456.58.

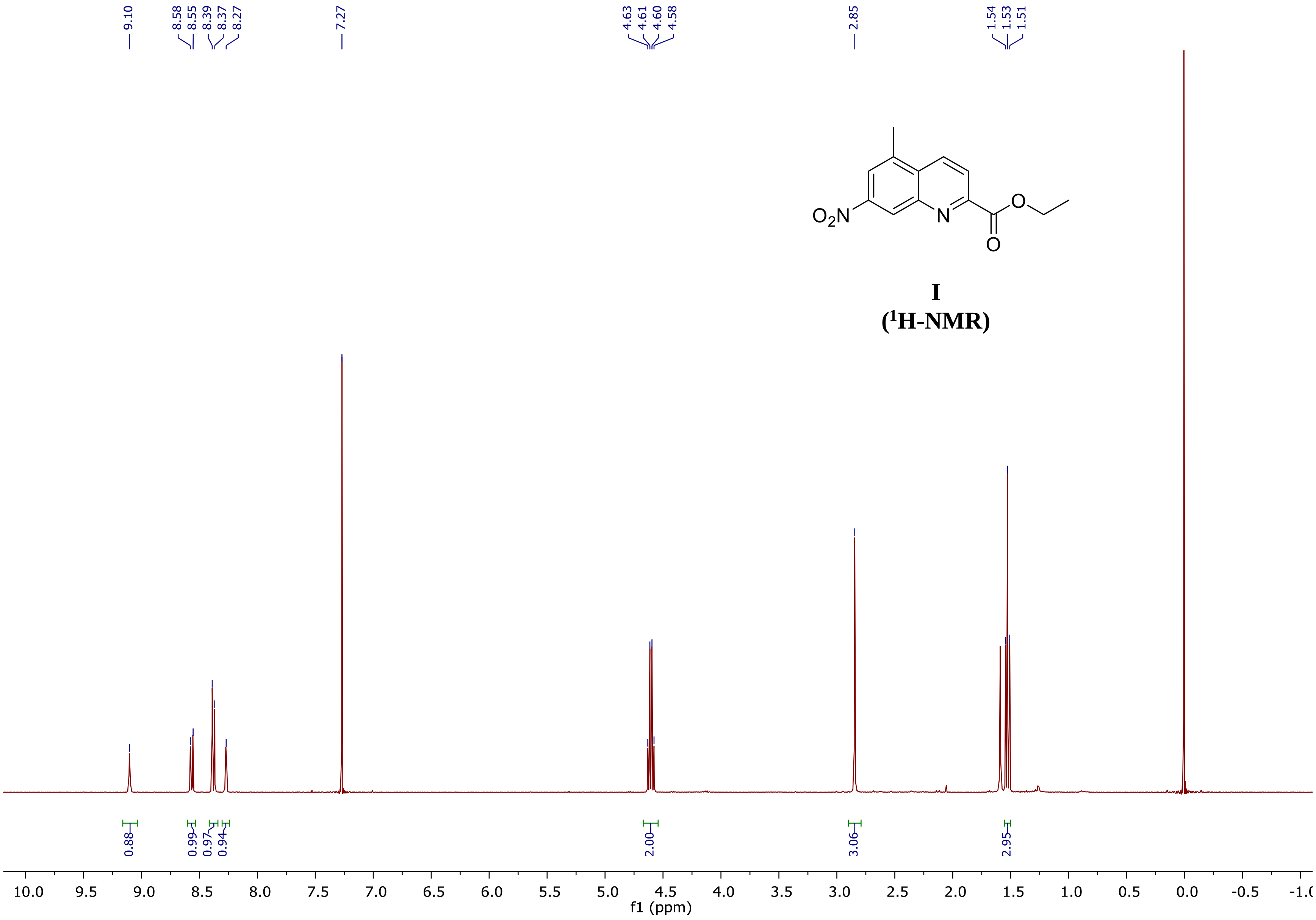
9.10
8.58
8.55
8.39
8.37
8.27
7.27
4.63
4.61
4.60
4.58
2.85
1.54
1.53
1.51
O2N
N
O
O
0.88
0.99
0.97
0.94
2.00
3.06
2.95
10.0
9.5
9.0
8.5
8.0
7.5
7.0
6.5
6.0
5.5
5.0
4.5
4.0
3.5
3.0
2.5
2.0
1.5
1.0
0.5
0.0
-0.5
f1 (ppm)

I
(^{1}H-NMR)

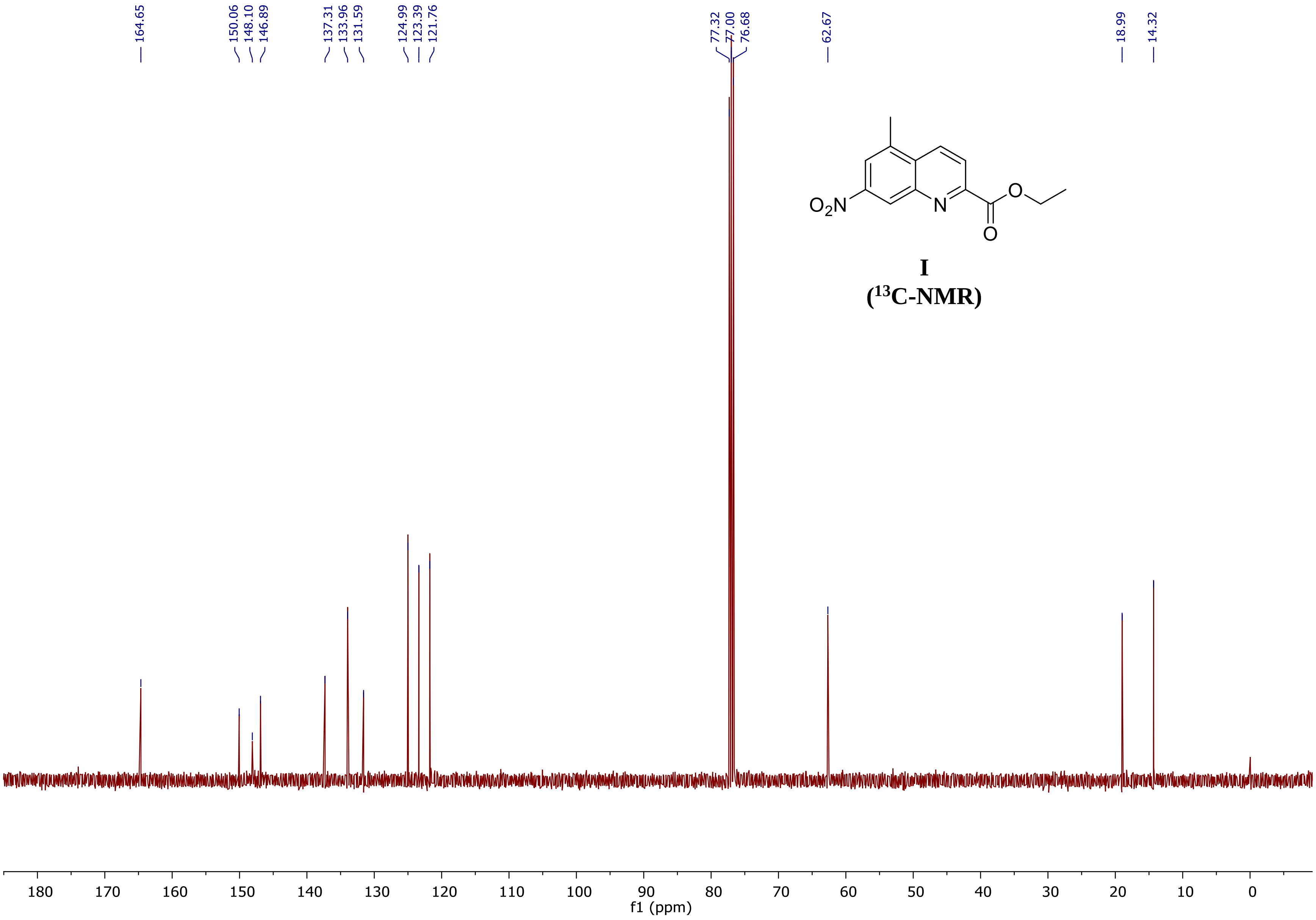
164.65
150.06
148.10
146.89
137.31
133.96
131.59
124.99
123.39
121.76
77.32
77.00
76.68
62.67
18.99
14.32
O2N
N
O
O
180
170
160
150
140
130
120
110
100
90
80
70
60
50
40
30
20
10
0
f1 (ppm)

I

(^{13}C-NMR)

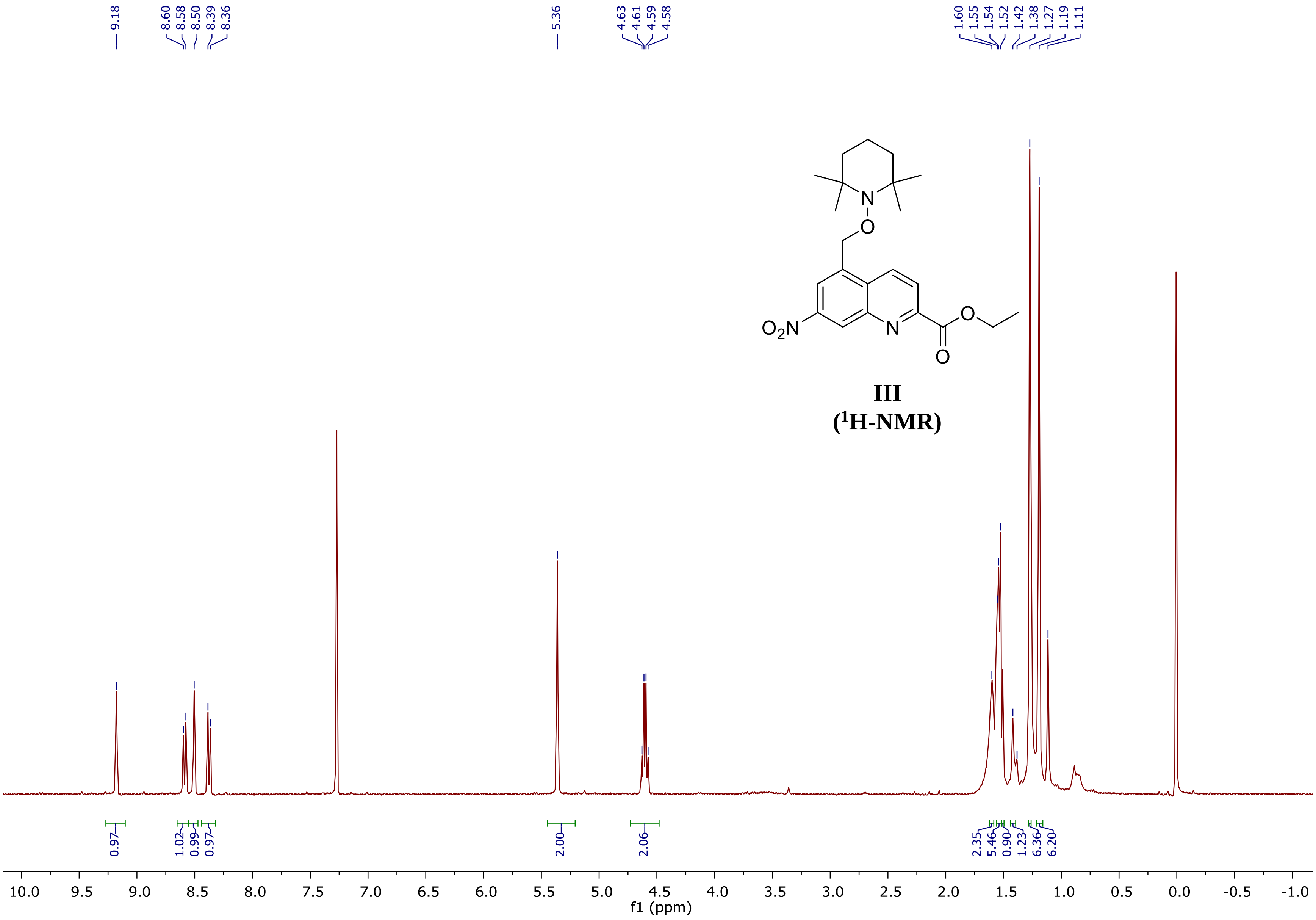

9.18
8.60
8.58
8.50
8.39
8.36
5.36
4.63
4.61
4.59
4.58
1.60
1.55
1.54
1.52
1.42
1.38
1.27
1.19
1.11
O_2N
N
O
III
(^{1}H-NMR)
0.97
1.02
0.99
0.97
2.00
2.06
2.35
5.46
0.90
1.23
6.36
6.20
10.0 9.5 9.0 8.5 8.0 7.5 7.0 6.5 6.0 5.5 5.0 4.5 4.0 3.5 3.0 2.5 2.0 1.5 1.0 0.5 0.0 -0.5 -1.0
f1 (ppm)

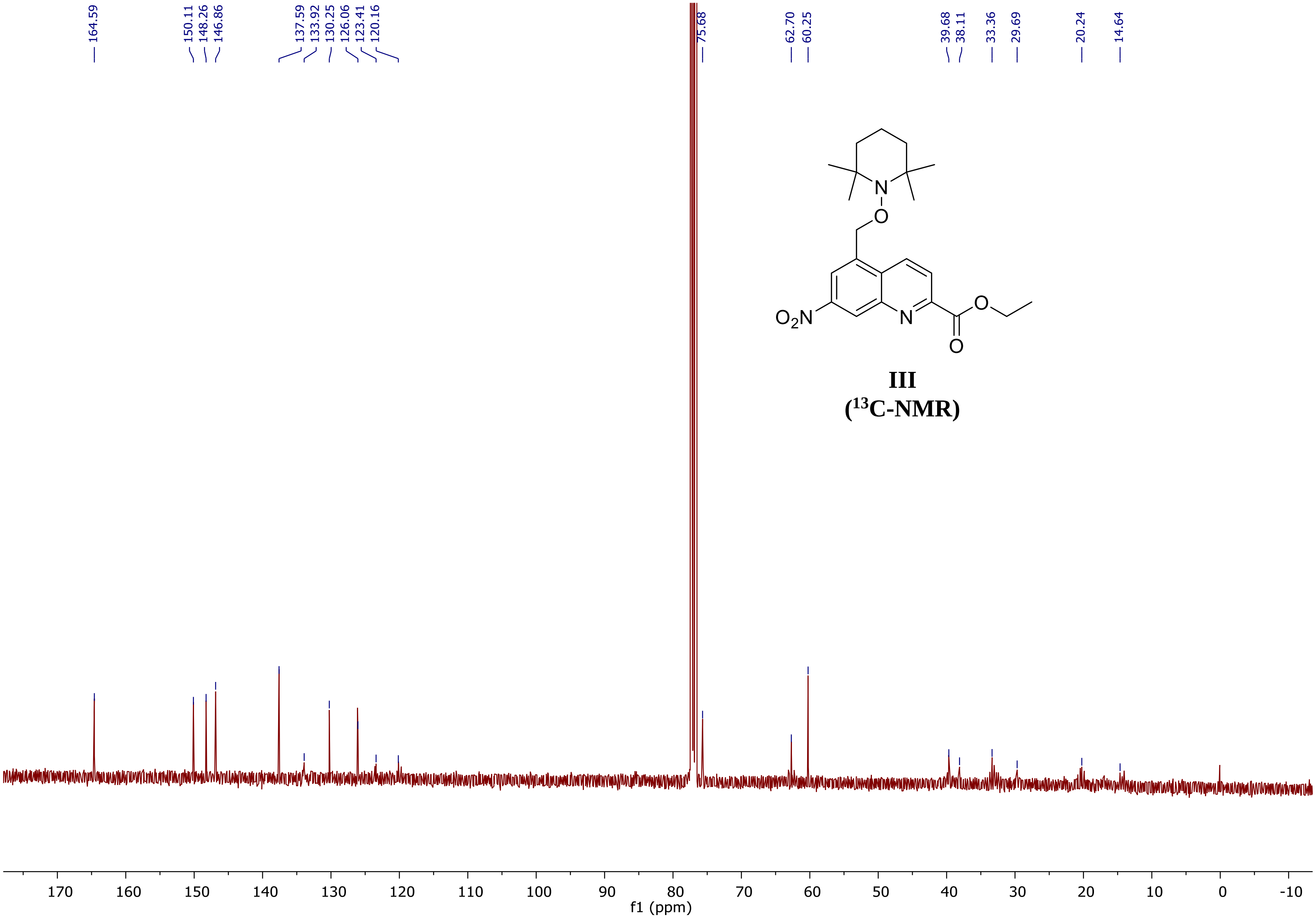
164.59
150.11
148.26
146.86
137.59
133.92
130.25
126.06
123.41
120.16
75.68
62.70
60.25
39.68
38.11
33.36
29.69
20.24
14.64
O2N
N
O
III
(13C-NMR)
f1 (ppm)

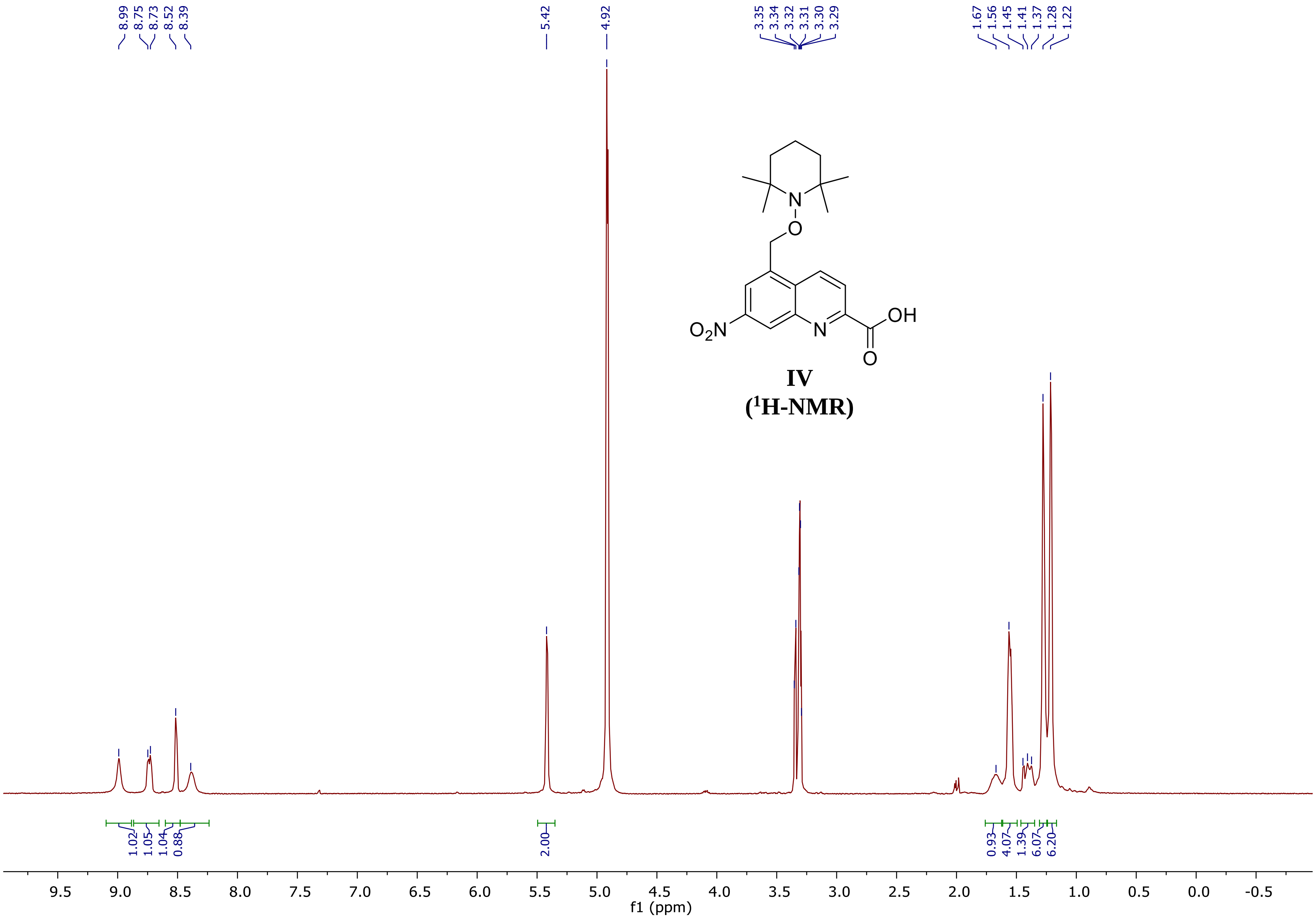

8.99
8.75
8.73
8.52
8.39
5.42
4.92
3.35
3.34
3.32
3.31
3.30
3.29
1.67
1.56
1.45
1.41
1.37
1.28
1.22
IV
(¹H-NMR)
1.02
1.05
1.04
0.88
2.00
0.93
4.07
1.39
6.07
6.20
f1 (ppm)

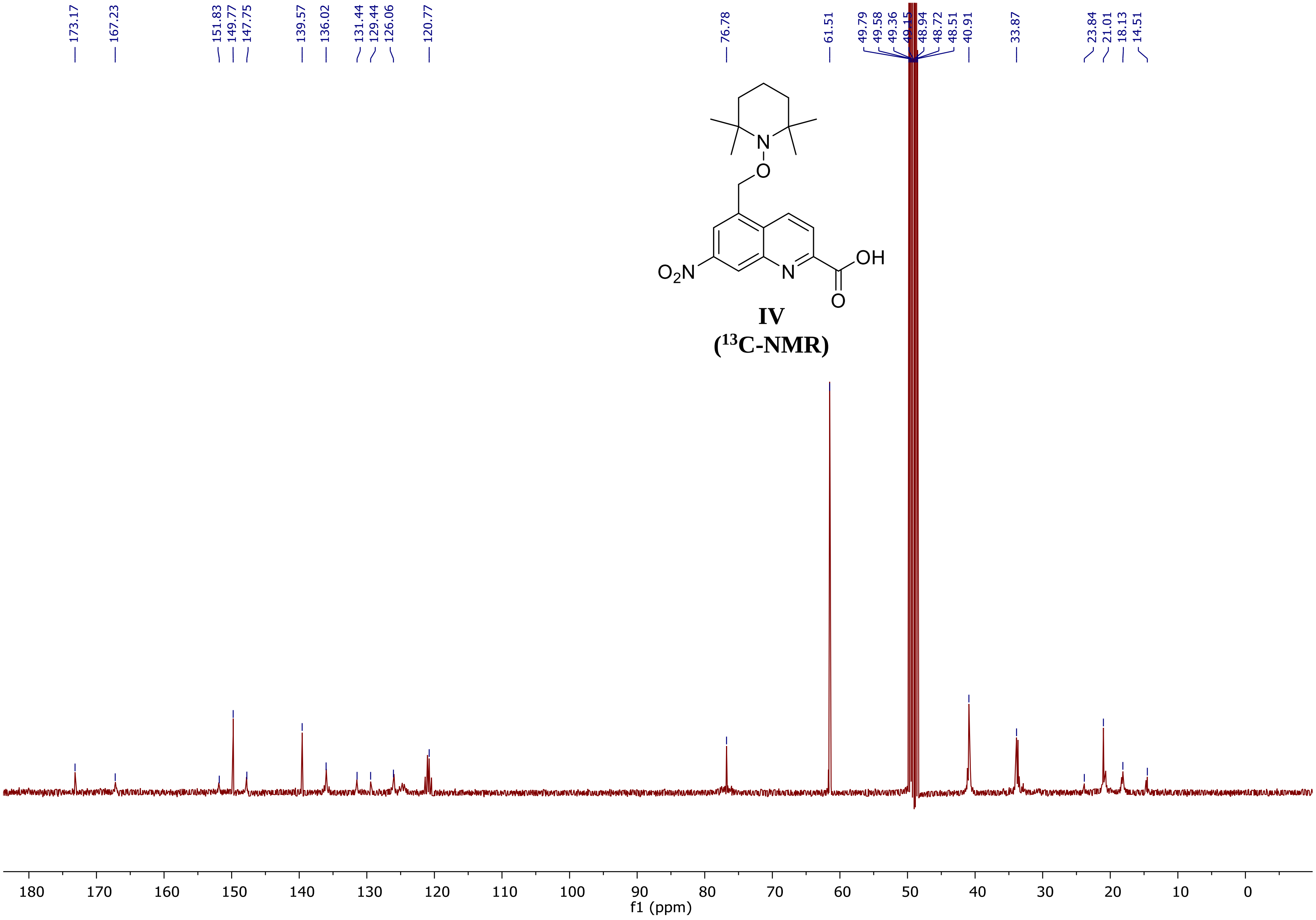
173.17
167.23
151.83
149.77
147.75
139.57
136.02
131.44
129.44
126.06
120.77
76.78
61.51
49.79
49.58
49.36
49.15
48.94
48.72
48.51
40.91
33.87
23.84
21.01
18.13
14.51
O2N
N
O
OH
N
O
IV
(13C-NMR)
180
170
160
150
140
130
120
110
100
90
80
70
60
50
40
30
20
10
0
f1 (ppm)

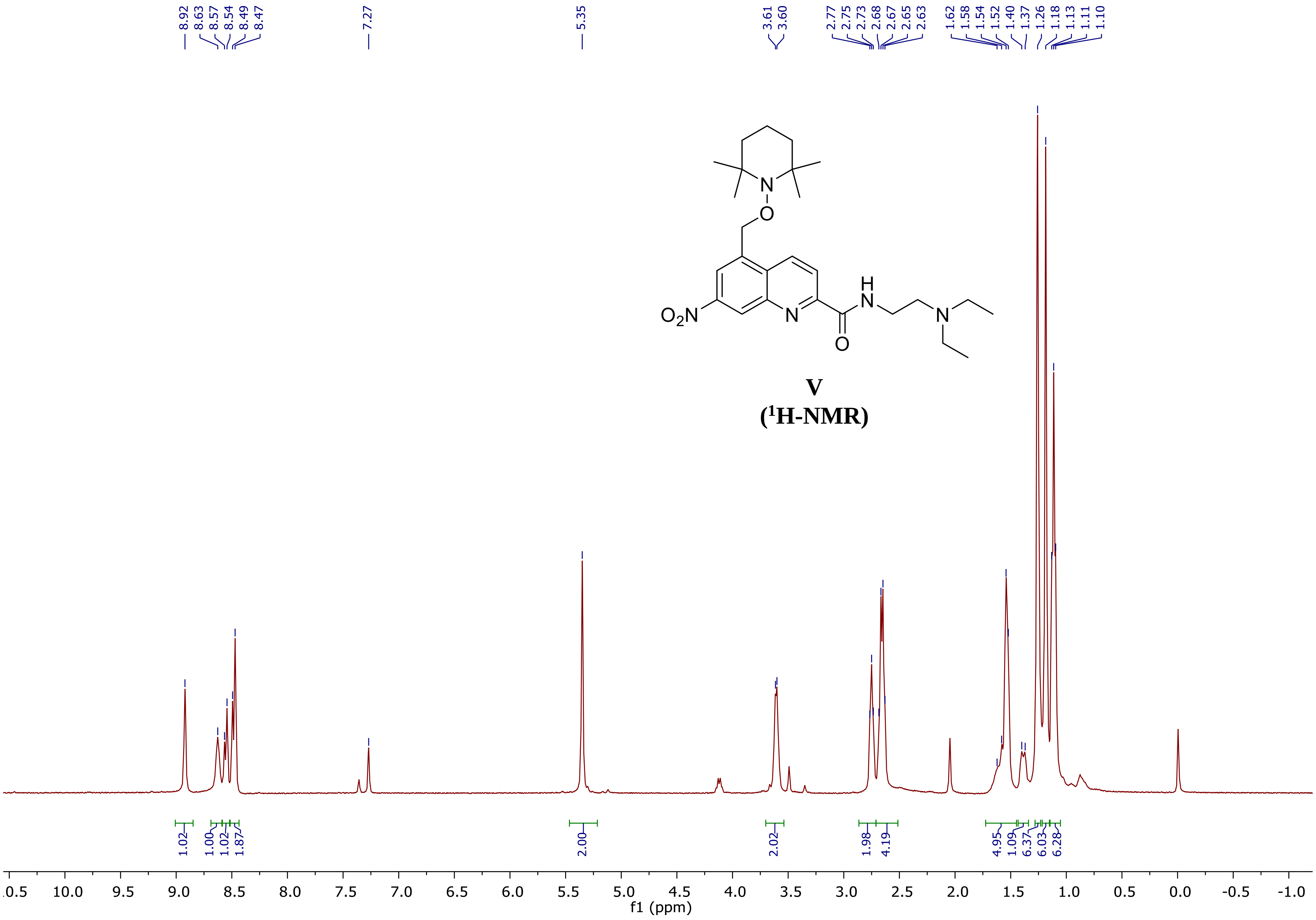
8.92
8.63
8.57
8.54
8.49
8.47
7.27
5.35
3.61
3.60
2.77
2.75
2.73
2.68
2.67
2.65
2.63
1.62
1.58
1.54
1.52
1.40
1.37
1.26
1.18
1.13
1.11
1.10
O_2N
N
H
N
O
N
N
O
1.02
1.00
1.02
1.87
2.00
2.02
1.98
4.19
4.95
1.09
6.37
6.03
6.28
f1 (ppm)

V
(^{1}H-NMR)

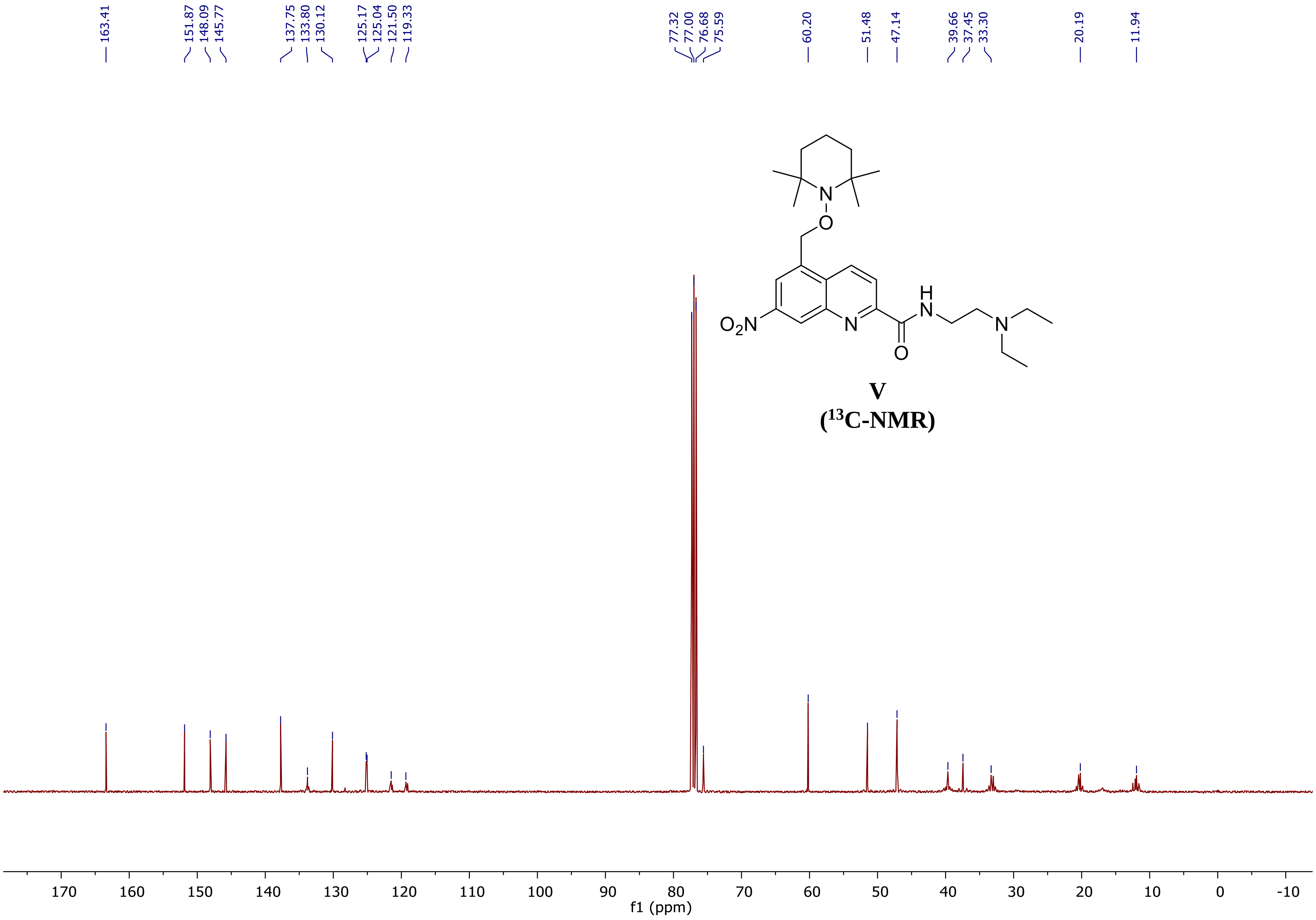
163.41
151.87
148.09
145.77
137.75
133.80
130.12
125.17
125.04
121.50
119.33
77.32
77.00
76.68
75.59
60.20
51.48
47.14
39.66
37.45
33.30
20.19
11.94
N
O
O_2N
N
H
N
N
O
V
(^{13}C-NMR)
170 160 150 140 130 120 110 100 90 80 70 60 50 40 30 20 10 0 -10
f1 (ppm)

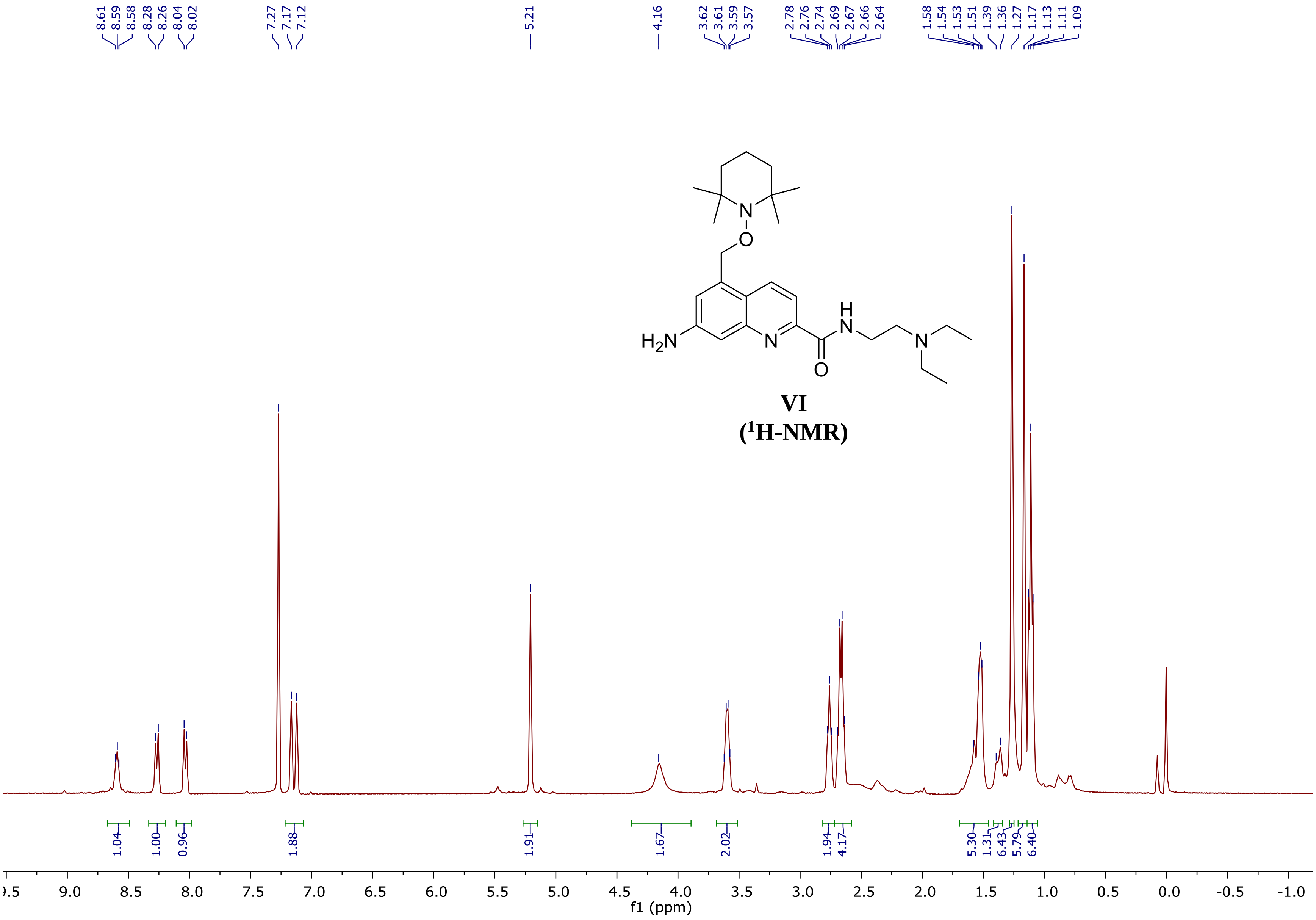
8.61
8.59
8.58
8.28
8.26
8.04
8.02
7.27
7.17
7.12
5.21
4.16
3.62
3.61
3.59
3.57
2.78
2.76
2.74
2.69
2.67
2.66
2.64
1.58
1.54
1.53
1.51
1.39
1.36
1.27
1.17
1.13
1.11
1.09
H$_2$N
N
O
N
H
N
O
N
VI
(^{1}H-NMR)
1.04
1.00
0.96
1.88
1.91
1.67
2.02
1.94
4.17
5.30
1.31
6.43
5.79
6.40
f1 (ppm)

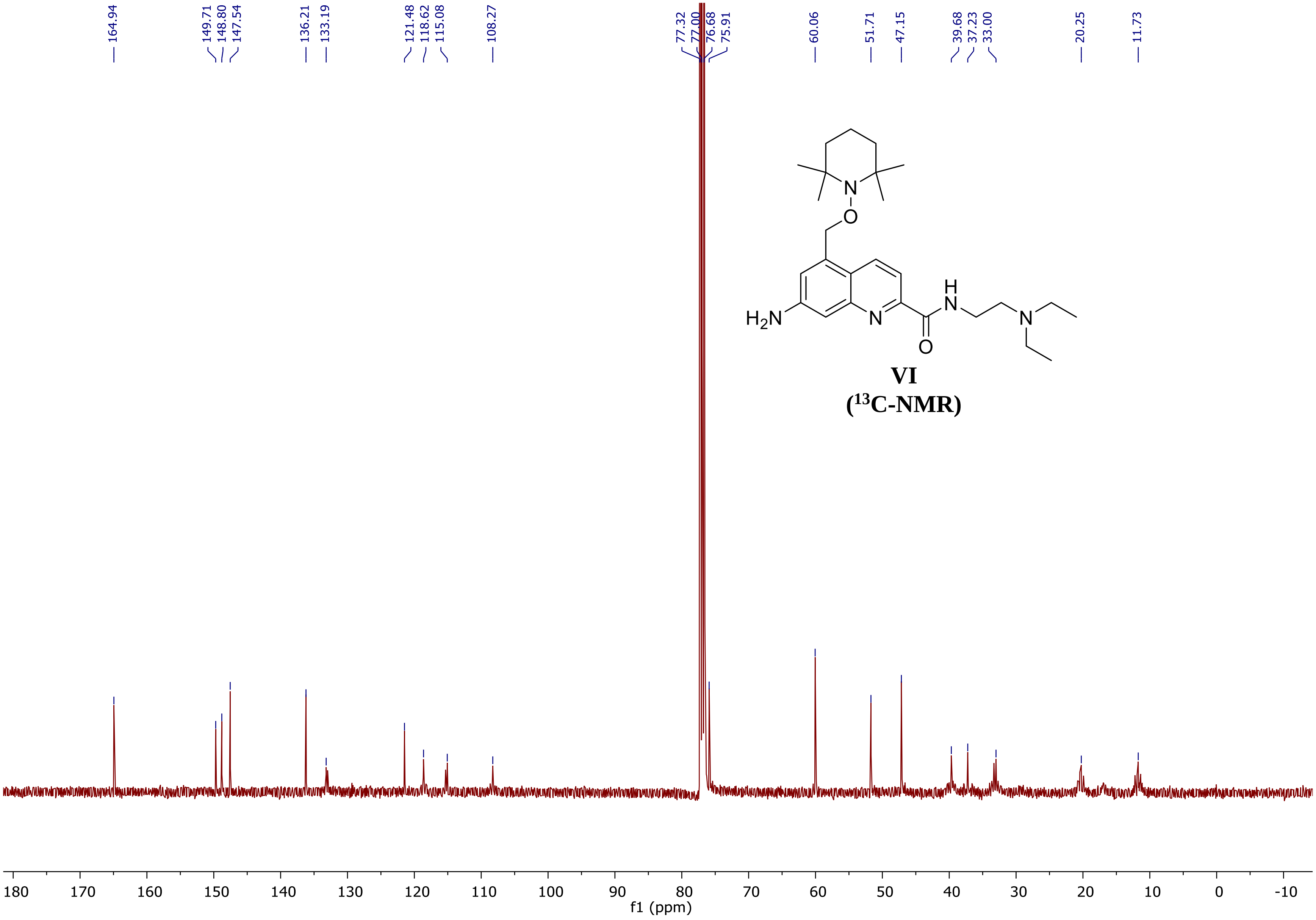
164.94
149.71
148.80
147.54
136.21
133.19
121.48
118.62
115.08
108.27
77.32
77.00
76.68
75.91
60.06
51.71
47.15
39.68
37.23
33.00
20.25
11.73
N
O
H
N
H_2N
N
N
O
f1 (ppm)

VI
(^{13}C-NMR)
